# Associations of ambient temperature exposure with embryonic and early fetal development

**DOI:** 10.1093/ije/dyag060

**Published:** 2026-05-24

**Authors:** Esmée Essers, Jasmin M de Groot, Naomi Graafland, Romy Gonçalves, Carmen Iñiguez, Sami Petricola, Vincent Jaddoe, Hanan El Marroun, Henning Tiemeier, Eric Steegers, Melek Rousian, Annemarie Mulders, Mònica Guxens

**Affiliations:** ISGlobal, Barcelona, Spain; Department of Medicine and Life Sciences, Universitat Pompeu Fabra, Barcelona, Spain; Department of Child and Adolescent Psychiatry/Psychology, Erasmus MC, University Medical Center, Rotterdam, The Netherlands; The Generation R Study Group, Erasmus University Medical Center, Rotterdam, The Netherlands; The Generation R Study Group, Erasmus University Medical Center, Rotterdam, The Netherlands; Department of Paediatrics, Sophia’s Children’s Hospital, Erasmus University Medical Center, Rotterdam, The Netherlands; The Generation R Study Group, Erasmus University Medical Center, Rotterdam, The Netherlands; Department of Obstetrics and Gynaecology, Erasmus MC, University Medical Center, Rotterdam, The Netherlands; The Generation R Study Group, Erasmus University Medical Center, Rotterdam, The Netherlands; Department of Paediatrics, Sophia’s Children’s Hospital, Erasmus University Medical Center, Rotterdam, The Netherlands; Spanish Consortium for Research on Epidemiology and Public Health (CIBERESP), Instituto de Salud Carlos III, Madrid, Spain; Department of Statistics and Operational Research, Universitat de València, Valencia, Spain; ISGlobal, Barcelona, Spain; The Generation R Study Group, Erasmus University Medical Center, Rotterdam, The Netherlands; Department of Paediatrics, Sophia’s Children’s Hospital, Erasmus University Medical Center, Rotterdam, The Netherlands; Department of Child and Adolescent Psychiatry/Psychology, Erasmus MC, University Medical Center, Rotterdam, The Netherlands; Department of Psychology, Education and Child Studies, Erasmus School of Social and Behavioural Sciences, Erasmus University Rotterdam, Rotterdam, The Netherlands; Department of Social and Behavioural Sciences, Harvard T.H. Chan School of Public Health, Boston, MA, United States; Department of Obstetrics and Gynaecology, Erasmus MC, University Medical Center, Rotterdam, The Netherlands; Department of Obstetrics and Gynaecology, Erasmus MC, University Medical Center, Rotterdam, The Netherlands; Department of Obstetrics and Gynaecology, Erasmus MC, University Medical Center, Rotterdam, The Netherlands; ISGlobal, Barcelona, Spain; Department of Medicine and Life Sciences, Universitat Pompeu Fabra, Barcelona, Spain; Department of Child and Adolescent Psychiatry/Psychology, Erasmus MC, University Medical Center, Rotterdam, The Netherlands; Spanish Consortium for Research on Epidemiology and Public Health (CIBERESP), Instituto de Salud Carlos III, Madrid, Spain; ICREA, Barcelona, Spain

**Keywords:** climate change, pregnancy, cohort study, environmental pollution, embryonic size, first trimester

## Abstract

**Background:**

Exposure to heat and cold are associated with adverse birth outcomes, but whether ambient temperature affects embryonic and early fetal development remains unclear. We aimed to examine the association between ambient temperature exposure during early pregnancy and crown–rump length (CRL).

**Methods:**

Data from the Generation R Next Study (2017–2021) were analysed, with findings replicated in the Generation R Study (2002–2006), both population-based cohorts based in Rotterdam, The Netherlands. Weekly mean temperatures were modeled from the last menstrual period onward at a spatial resolution of 100 × 100 m by using the UrbClim™ model. The CRL was measured via 2D ultrasound at approximately 8, 10, and 12 weeks’ gestation in pregnancies with regular menstrual cycles. Distributed lag nonlinear models were applied.

**Results:**

In Generation R Next (*N* = 1378; mean maternal age 31.9 years), higher temperatures during the first 9 weeks were associated with a smaller CRL at 12 weeks {e.g. −7.2 mm [95% confidence interval (CI) −12.0, −2.3] at 19.2 vs 9.0°C during weeks 1–6}. Colder exposures during the first 11 weeks were also associated with a smaller CRL [−7.6 mm (95% CI −11.9, −3.3) at 3.6 vs 9.0°C during weeks 1–11]. No associations were observed for CRL at 8 or 10 weeks. Similar associations with cold, but not heat, were observed in the replication cohort (*N* = 1520).

**Conclusion:**

Moderate cold and heat exposure during early pregnancy may affect fetal development as early as the first trimester. These findings indicate that early gestational development may be sensitive to ambient temperature and, as environmental conditions shift, may have potential clinical implications for birth outcomes and long-term health.

Key Messages

- We sought to investigate the effects of early-pregnancy ambient temperature exposure on early fetal development.
- In a cohort of pregnant individuals in the Netherlands, we found that moderate heat and cold during the first trimester were associated with a smaller crown–rump length (CRL) at 12 weeks. In a replication cohort, similar associations were observed for cold.
- Early-pregnancy exposure to moderate cold and heat is associated with a smaller CRL, suggesting that climate change may influence early development.

## Introduction

In recent years, frequent, intense, and prolonged exposure to temperature extremes has increased [1, 2]. While climate change affects all individuals [3, 4], pregnant women are particularly susceptible due to physiological changes during pregnancy [5–8]. It is hypothesized that temperature extremes may disrupt maternal thermoneutrality [6, 9], which can lead to placental dysregulation through oxidative stress, heat shock protein activation, and changes in uteroplacental blood flow, impairing nutrient and oxygen transfer to the offspring and potentially compromising development [6, 7, 9, 10].

Epidemiological studies have found that exposure to cold and heat is associated with pregnancy complications and adverse birth outcomes [5, 9, 11]. Additionally, two studies observed associations with changes in fetal size or growth metrics in mid- and late pregnancy [12, 13]. However, to the best of our knowledge, no study has examined the effects on embryonic and early fetal development. The first trimester is critical for organogenesis and placentation [14, 15]. Suboptimal development during this stage, often assessed through deviations in the crown–rump length (CRL) as a measure of embryonic and early fetal size, has been associated with adverse birth outcomes as well as long-term cardiovascular and respiratory effects in childhood [16, 17]. Therefore, we aimed to evaluate the association between ambient temperature exposure and embryonic and early fetal development in a Dutch prospective birth cohort. We also aimed to replicate the findings in an independent cohort also from the Netherlands, established 15 years earlier.

## Methods

### Study population and design

This study is embedded in the Generation R Next Study—a population-based birth cohort based in Rotterdam, The Netherlands [18]. Women aged ≥18 years, resident in Rotterdam, and attempting to conceive or pregnant were enrolled from preconception onward. Between August 2017 and July 2021, 4033 preconception or pregnancy episodes from 3602 women were included (Supplementary Figure 1). The current study includes women with at least one first-trimester CRL measurement, gestational age ≤13.9 weeks [19], regular menstrual cycle and known first day of the last menstrual period, and complete temperature data for the first 6 weeks of pregnancy (*N* = 1378) (Supplementary Figure 1). The CRL was collected at three intended first-trimester time points, with mean gestational ages of 7.6 (range 4.6–10.6), 9.6 (6.7–10.6), and 12.0 (9.0–13.9) weeks, referred to as 8-, 10-, and 12-week measurements. To align the assigned time points with actual gestational age, we recategorized the measurements as follows: ≤9.0 weeks as the 8-week measurement group, >9.0 to ≤11.0 weeks as the 10-week measurement group, and >11.0 weeks as the 12-week measurement group (Supplementary Figure 2). If a participant contributed more than one measurement within a group, then the measurement closest to that group’s median gestational age was retained. The sample sizes reflected availability of temperature and outcome data: temperature during weeks 1–6 and CRL at 8 weeks (*N* = 876), temperature during weeks 1–9 and CRL at 10 weeks (*N* = 921), and temperature during weeks 1–11 and CRL at 12 weeks (*N* = 1160). The temperature-exposure windows maximized duration while minimizing data loss.

### Temperature exposure

Ambient air temperature was modeled by using the UrbClim^TM^ model (VITO, Belgium) [20]. This urban climate model simulates interactions between urban surfaces and the atmosphere, incorporating detailed land-use and urban-structure information alongside a simplified representation of the lowest part of the atmosphere where most weather and human exposure occurs (Supplementary Methods S1). Hourly temperature data (°C) were estimated at a height of 2 m above ground level with a spatial horizontal resolution of 100 × 100 m. Temperature data were assigned to each participant’s address during pregnancy, considering any address changes. The mean temperatures for each pregnancy week were calculated: 7 consecutive days starting from the first day of the last menstrual period. Validation of the UrbClim^TM^ data (daily data for 2017–2020) was performed against daily E-OBS European temperature data and showed high performance (multiple *R*^2^ of 0.98 and root mean squared error of 0.9°C) [21].

### Embryonic and early fetal outcome

As CRL was the study outcome, gestational age was calculated from the first day of the last menstrual period [22]. The gestational age was considered unreliable if the average cycle length was unknown or irregular (<21/>35 days). Information on menstrual cycle and last menstrual period was collected through questionnaires during early pregnancy and confirmed at ultrasound visits [14, 18].

During the first pregnancy trimester, trained sonographers performed transvaginal 2D-ultrasound examinations following cohort-specific guidelines [23–26]. Training was undertaken by following national standards (Dutch Ministry of Public Health, Welfare, and Sport), thus the measurement variability was assumed to be representative of clinical practice in the Netherlands. Ultrasound imaging was conducted by using the Philips© Affiniti 70, Philips© Epiq 7, or Voluson© E10 systems. Transducers with varying frequencies (MHz) were selected based on the highest-quality imaging. As a metric for embryonic and early fetal size, the CRL was measured in a straight line within the mid-sagittal plane as the greatest length from the cranium to the caudal rump (millimeters), taking the mean of three measurements [27].

### Potential confounding variables

Considering the availability of data, previous literature, and biological plausibility, potential confounding variables were identified a priori by using a directed acyclic graph (Supplementary Figure 3). Information relating to the fetus (biological sex), the parents (age at recruitment, national origin, education level), the residence (partnered or cohabiting), monthly net household income, and the mother (parity, alcohol consumption, smoking habit, folic acid supplement use) was included. The parental body mass index (kg/m^2^) was calculated by using the self-reported or measured pre-pregnancy height and weight. The residential surrounding greenness levels were estimated for each exposure period within a buffer of 300 m by using the Normalized Difference Vegetation Index [28]. The neighborhood socioeconomic status of the residential address during each exposure period was derived by using Statistics Netherlands data [29]. We considered seasonality through the month of conception.

### Statistical analyses

We imputed missing values of the potential confounding variables to reduce potential selection bias by using expectation-maximization imputation (Supplementary Table 1) [30]. The percentage of missing values for all variables was <18% and the imputed and observed datasets had similar distributions (Supplementary Table 2). Pregnant women included and excluded in the analysis sample had some different population characteristics (Supplementary Table 3). Thus, inverse probability weighting was performed in the sample including all singleton pregnancies to reduce selection bias (Supplementary Table 4 and Supplementary Figure 4) [31].

We evaluated associations between weekly first-trimester ambient temperature and CRL outcomes separately by using distributed lag nonlinear models (DLNMs) within a generalized linear model framework [32]. This approach estimates nonlinear exposure–response relationships while accounting for delayed (lagged) effects of exposure over time through the lag–response structure, introducing an additional model dimension representing temporal dependency across exposure weeks. Each pregnancy week was defined as one lag. We ran one model for the CRL at 8 weeks (lags of 1–6 weeks), two for the CRL at 10 weeks (lags of 1–6 and 1–9 weeks), and three for the CRL at 12 weeks (lags of 1–6, 1–9, and 1–11 weeks). In the models, natural cubic splines (two knots at the 25th/75th percentiles) for exposure–response and linear splines for lag–response were used. The results are shown in reference to the temperature at which the largest CRL effects were observed across the outcomes (4.3°C, 22.4°C, and 9.0°C for 8-, 10-, and 12-week CRLs, respectively). The models were fitted by using the “dlnm” v2.4.7 R-package and glm() function. Final estimates and 95% confidence intervals (CIs) are presented as the cumulative effect of temperature exposure for each lag period (1–6, 1–9, or 1–11 weeks) on each CRL outcome (at 8, 10, or 12 weeks). Cumulative effects were derived by using the crossreduce() function of the “dlnm” R-package, which sums the lag-specific contributions to produce an exposure–response curve for each exposure period and outcome. Models were adjusted for the abovementioned potential confounding variables and the gestational age at each outcome assessment, with multiple testing correction applied by using the effective number of tests approach (*α* = 0.025).

To assess the robustness of the results, we performed sensitivity analyses. First, for associations surviving multiple testing correction, lag–response curves were used to estimate the effect of exposure to cold and hot temperatures (depending on which temperatures were identified) across each pregnancy week. Second, DLNMs were rerun restricting to participants with CRL measurements at all three time points (*N* = 495). Third, the CRL growth was evaluated (see Supplementary Methods S2). Fourth, associations for shorter exposure windows were extracted from models including longer-lag structures to assess potential bias from correlated exposure across weeks. Finally, analyses were repeated with alternative DLNM parameterizations. Analyses were performed by using R v4.3.2 (R Core Team 2023).

### Replication study

We used data from the Generation R Study—an earlier (April 2002 to January 2006) population-based prospective cohort from fetal life onward based in Rotterdam, The Netherlands that included 7145 pregnant women [33]. CRL measurements via ultrasound were taken at a mean gestational age of 12.4 weeks (range 11.0–13.9) (*N* = 1520). Temperature exposure was estimated by using the same UrbClim™ model. All data processing and DLNM analyses followed the same specifications as the main analysis. Additionally, we performed a meta-analysis combining cohort-specific DLNM estimates (main and replication cohort) via random effects modeling fitted through restricted maximum likelihood, including a cohort-level random effect.

## Results

**Figure 1:**
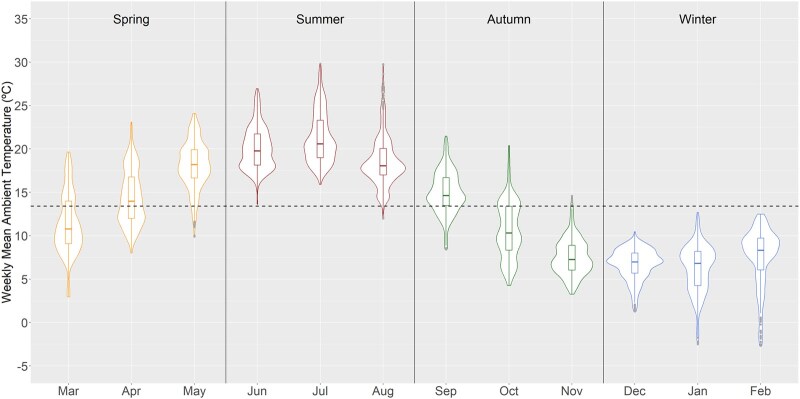
Distribution of mean weekly ambient temperature by month during weeks 1–6 of pregnancy (*N* = 1378). Temperature exposure is for the years 2017–2020. The width of the violin plots indicates the density of the temperature observations at that °C value. The box plots within the violin plots show the median and interquartile ranges, with the upper and lower boundaries indicating the maximum and minimum temperature values, respectively. The dashed horizontal line indicates the overall mean temperature at 13.4°C.

Pregnant women were on average 31.9 years old (SD 3.9) and parents were mostly from the Netherlands (pregnant women 66.2%, partners 64.9%) (Table 1). The mean weekly ambient temperature during pregnancy weeks 1–6 was 13.4°C (1st–99th percentiles 2.0–26.2°C) (Fig. 1). The average CRL at 8 weeks was 14.1 mm (SD 5.2), 31.5 mm (SD 7.8) at 10 weeks, and 58.3 mm (SD 8.9) at 12 weeks. In the replication cohort, the mean temperature was 11.3°C (1st–99th percentiles −0.7°C to 23.9°C) (Supplementary Figure 5). The average CRL was 61.9 mm (SD 10.6) at 12 weeks.

**Table 1:** Distribution of population characteristics.

	Participants (*N* = 1378)
**Fetal characteristics**	
Sex (female vs male) [*n* (%)]	653 (47)
Season of conception [*n* (%)]	
Summer	376 (27)
Autumn	385 (28)
Winter	269 (20)
Spring	348 (25)
**Maternal characteristics**	
Age at recruitment (years) [mean (SD)]	31.9 (3.9)
Body mass index (kg/m^2^) [mean (SD)]	24.3 (4.4)
National origin [*n* (%)]	
The Netherlands	895 (66)
Suriname/Dutch Caribbean	141 (10)
Other global south countries^a^	177 (13)
Other global north countries^b^	139 (10)
Educational level [*n* (%)]	
High	982 (73)
Medium	304 (23)
Low	52 (4)
Parity [*n* (%)]	
No children	856 (65)
1 child	359 (27)
≥2 children	107 (8)
Smoking habit [*n* (%)]	
Did not smoke	683 (56)
Quit smoking before pregnancy	382 (31)
Smoked during pregnancy	163 (13)
Alcohol consumption [*n* (%)]	
No consumption <3 months before pregnancy	257 (20)
Consumption <3 months before pregnancy	834 (64)
Consumption during pregnancy	215 (17)
Folic acid use [*n* (%)]	
Started use prior to pregnancy	906 (70)
Did not use or started during pregnancy	392 (30)
**Partner characteristics**	
Age at recruitment (years) [mean (SD)]	33.9 (5.1)
Body mass index (kg/m^2^) [mean (SD)]	25.6 (3.7)
National origin [*n* (%)]	
The Netherlands	842 (65)
Suriname/Dutch Caribbean	124 (10)
Other global south countries^a^	215 (17)
Other global north countries^b^	117 (9)
Educational level [*n* (%)]	
High	831 (64)
Medium	371 (29)
Low	99 (8)
**Residential characteristics**	
Residential surrounding greenness [mean (SD)]^c^	0.4 (0.1)
Neighborhood socioeconomic status [mean (SD)]^c^	–0.1 (0.1)
Partner/co-habiting (yes vs no) [*n* (%)]	1162 (89)
Monthly net household income [*n* (%)]	
<€2000	116 (10)
€2000–4000	387 (32)
€4000–6000	525 (44)
>€6000	178 (15)

a Countries categorized as global south include Turkey, Morocco, Cape Verde, Indonesia, China, countries in Africa, other American and Asian non-Western countries.

b Countries categorized as global north include Germany, Yugoslavia, Poland, other American and Asian non-Western countries, other European and all Oceania countries.

c Exposure calculated from the date of last menstrual period until the date of the 8-week ultrasound.

**Figure 2:**
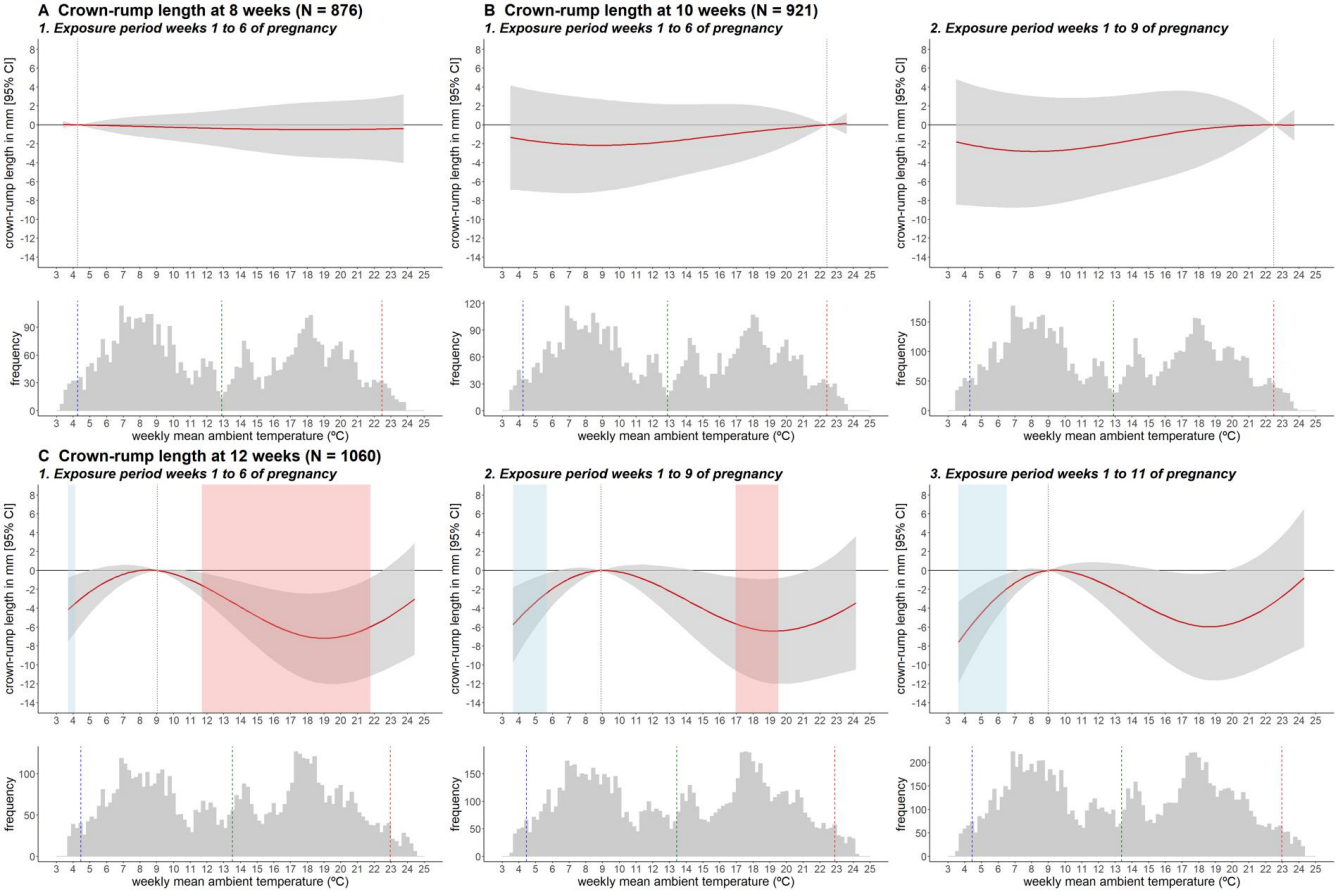
Cumulative associations between weekly ambient temperature exposure for different exposure periods and CRL at 8, 10, and 12 weeks of pregnancy and the respective distributions of temperature. The *x*-axes depict the 3rd to 97th percentiles of the temperature distribution: (a) 3.4°C–23.9°C, (b1) 3.5°C–23.7°C, (b2) 3.4°C–23.7°C, (c1) 3.7°C–24.4°C, (c2) 3.6°C–24.2°C, (c3) 3.6°C–24.3°C. In the upper plots, the solid curved lines (in red) represent the associations derived from the DLNMs, expressed as beta coefficients of the CRL with their respective 95% confidence intervals (CIs) shown as shaded bands (in gray). Coefficients are estimated as the change in CRL in millimeters at each temperature with respect to the reference temperature [vertical dotted lines (in black), 4.3°C for (a), 22.4°C for (b), and 9.0°C for (c)]. Shaded areas to the left (in blue) and right (in red) of the reference temperatures (exposure to colder or warmer temperatures with respect to the reference temperature) indicate associations surviving correction for multiple testing (*P* < .025). The lower plots represent the distribution of temperatures, with the dotted vertical lines indicating the 5th percentile, mean, and 95th percentile of temperature distribution (from left to right in blue, green, and red). DLNMs were adjusted for fetal biological sex; parental age at recruitment, national origin, educational level, and body mass index; parents partnered or cohabiting, monthly net household income, maternal parity, alcohol consumption, smoking habit, folic acid use, and month of last menstrual period; residential surrounding greenness, neighborhood socioeconomic status, and gestational age at the ultrasound.

Temperature exposure during the first 6 weeks of pregnancy and the CRL at 8 weeks revealed null associations (Fig. 2a, Supplementary Table 5, and Supplementary Figure 6). Similarly, the cumulative associations between temperature during the first 6 and 9 weeks of pregnancy and the CRL at 10 weeks were close to the null (Fig. 2b, Supplementary Table 5, and Supplementary Figure 6). For CRL at 12 weeks, exposure to warmer temperatures was associated with smaller CRLs, particularly for shorter exposure periods (Fig. 2c, Supplementary Table 5, and Supplementary Figure 6). Specifically, cumulative exposures between 11.7°C and 21.8°C (in reference to 9.0°C) during pregnancy weeks 1–6 and between 17.0°C and 19.5°C during weeks 1–9 were associated with a smaller CRL at 12 weeks [e.g. 7.2 mm smaller (95% CI −12.0, −2.3) for exposure to 19.2°C during weeks 1–6]. Moreover, exposure to colder temperatures for all exposure periods was associated with a smaller CRL (Fig. 2c, Supplementary Table 5, and Supplementary Figure 6). Specifically, cumulative exposure between 2.1°C and 4.1°C during pregnancy weeks 1–6, 2.1°C to 5.7°C during weeks 1–9, and 2.0°C to 6.5°C during weeks 1–11 (in reference to 9.0°C) was associated with a smaller CRL at 12 weeks [e.g. 7.6 mm smaller (95% CI −11.9, −3.3) for exposure to 3.6°C during weeks 1–11]. The lag–response curves showed that, for heat exposure (19.1°C vs 9.0°C), the first 6 weeks were the most susceptible [e.g. 2.4 mm smaller (95% CI −3.8, −1.0) for exposure to 19.1°C during week 1], whereas, for cold exposure (3.6°C vs 9.0°C), weeks 2–9 were susceptible [e.g. 0.8 mm smaller (95% CI −1.5, −0.1) for exposure to 3.6°C during week 2] (Supplementary Figure 7).

Sensitivity analyses restricting DLNMs to participants with CRL at all three time points and evaluating CRL growth showed similar curves, though no associations were identified (Supplementary Figures S8 and S9). Associations for shorter lag periods derived from longer-lag models were nearly identical (Supplementary Figure 10). Alternative DLNM parameterizations produced comparable results (Supplementary Figures S11–S14).

Replication in Generation R and meta-analysis confirmed associations between cold exposure and smaller CRLs at 12 weeks, particularly for shorter exposure periods, while associations for warmer temperatures were close to the null (Supplementary Figures S15 and S16).

## Discussion

Exposure to colder and warmer ambient temperatures during the first trimester of pregnancy was associated with smaller CRLs at 12 weeks in a Dutch cohort study. No associations for cold or heat exposure during early pregnancy with CRLs at 8 or 10 weeks were found. The association of cold exposure and smaller CRLs at 12 weeks replicated that in an independent cohort established 15 years earlier in the same region, in which participants were exposed to colder temperatures than the primary cohort. Differences in the timing and strength of associations between the cohorts may reflect variations in climate patterns, population-level vulnerability, or adaptive responses over time.

While previous research has demonstrated that exposure to cold (e.g. <1st percentile) and heat (e.g. temperature increases or heatwaves) during pregnancy increases the risk of adverse birth outcomes, the potential impacts on embryonic and fetal development remain less understood [5, 11]. Epidemiological studies, including our earlier work in Generation R, have identified associations between cold or heat exposure and changes in fetal growth in mid- and late pregnancy [12, 13]. Additionally, cold and heat stress during the second and third trimesters in mice (equivalent to pregnancy week 12 onward in humans) was associated with smaller CRLs [34]. However, to our knowledge, this is the first study to have examined the relationship between temperature exposure during the first trimester and embryonic and early fetal development in humans. Physiological changes in pregnancy challenge thermoregulation and external temperature stress may further disrupt this balance, potentially compromising placental development [35]. Heat and cold exposure can trigger thermoregulatory responses, such as altered peripheral blood flow, which may reduce uterine perfusion and interfere with processes such as spiral artery remodeling [36–38]. Disruption of these early placental processes could impair oxygen and nutrient exchange between mother and child, ultimately affecting early development [7, 36]. In addition, preconception temperature exposure may influence gametogenesis in both parents, including heat-related reductions in sperm quality and the disruption of oocyte maturation [39, 40]. Such upstream influences could contribute to developmental changes observed in early gestation, even before implantation or placentation.

The magnitude of the associations observed between ambient temperature exposure and embryonic and early fetal development is noteworthy. Between July 2017 and October 2020, the average winter temperature in the Netherlands was 4.2°C [41]. Exposure to colder-than-average conditions, such as 3.6°C during pregnancy weeks 1–11, was associated with a 7.6-mm smaller CRL at 12 weeks. To contextualize this, the INTERGROWTH-21st standards estimate an average CRL of 58 mm at 12 weeks [42], aligning with our 57-mm mean. A 7.6-mm reduction represents a 13.3% smaller-than-expected size at this developmental stage. Similarly, during summer, when the average temperature was 18.3°C, exposure to slightly elevated conditions (e.g. 19.2°C during weeks 1–6) was associated with a 7.2-mm smaller CRL, which is a 12.6% decrease. The effects of heat and cold fall within a range of biological relevance, particularly during a period of rapid embryonic growth. We observed associations with moderate heat (11.7°C–21.8°C) and less precise estimates at higher temperatures, likely due to limited data at the upper tail of the temperature distribution. Notably, the 99th percentile of temperature was 26.3°C, representing a moderate level of heat in the broader context of extreme-heat studies. While the DLNM cumulative estimates assume constant exposure across weeks, the moderate temperatures observed suggest that such multi-week exposure scenarios are not implausible. Especially under projected climate-change scenarios, pregnant populations are expected to experience higher and more prolonged heat exposures [43]. Considering the emerging effects suggested by the contrasts between our primary cohort and data from 15 years earlier, future pregnant women may face greater risks.

The effects of cold and heat exposure on the CRL were most evident at 12 weeks of gestation, but across different exposure windows. The heat effects were strongest during early pregnancy (weeks 1–6), while the cold effects were more pronounced over a longer period (weeks 1–11). This pattern likely reflects how cumulative effects in DLNMs integrate temperature across lags, with longer windows potentially diluting or amplifying associations depending on later-week contributions. Results suggest that early pregnancy is sensitive to heat, as supported by the lag–response curves highlighting weeks 1–6, whereas cold may influence CRLs over a longer period. The absence of associations for CRLs at 8 or 10 weeks may reflect the difficulty of detecting effect sizes earlier in pregnancy, when CRL measurements are smaller in absolute terms. Similarly, no associations were detected for CRL growth, consistently with the null effects at 8 and 10 weeks. This could reflect attenuation from aggregating early measurements or the limited exposure window. Associations observed only at 12 weeks suggest that temperature-related disruptions may not immediately impair early development, but could interfere with critical physiological processes occurring near the end of the first trimester. Spiral artery remodeling and the onset of maternal blood flow into the placenta, which are essential for adequate placental perfusion, occur at around this time and disruption may limit fetal growth potential [36, 38, 44]. Utero–placenta vascular development may represent an important pathway through which early temperature-exposure influences first-trimester development [36]—a critical window for organogenesis and placental development that lays the foundation for lifelong health [16, 45–47].

The key strength of this study is the unique and extensive first-trimester ultrasound data from a large, population-based birth cohort and the inclusion of a replication cohort. CRL data were collected by using advanced high-resolution ultrasound machines, following a standardized protocol, ensuring high-quality images to determine embryonic and early fetal size. Additionally, we leveraged high-resolution temperature data, accounting for potential address changes to reduce exposure misclassification. Lastly, the use of DLNMs enabled the assessment of cumulative temperature effects across pregnancy, incorporating time-series correlations and modeling heat and cold effects effectively.

Several limitations warrant consideration. First, exposure misclassification cannot be ruled out, as the ambient temperature was estimated from residential addresses, without accounting for individual-level modifiers such as housing or behaviors (e.g. time outdoors, heating/cooling systems). Despite the adjustment for neighborhood socioeconomic status, greenness, and household composition, residual confounding remains possible. Gestational age misclassification may have occurred due to cycle variability or recall bias, potentially overestimating the gestational age, especially in cases of delayed ovulation [48]. Early ultrasound dating is generally more precise [19], but using it would introduce circularity, as the CRL informs the ultrasound dating algorithm [49]. Nevertheless, the CRL is a sensitive and standardized indicator of early development, with excellent reproducibility when measured by trained sonographers. To reduce misclassification, only women with regular cycles and reliable last-menstrual-period information were included. Finally, DLNMs require complete exposure histories, so the exposure periods were predefined to maximize inclusion, potentially missing exposure weeks closer to the CRL measurement.

## Conclusion

With climate projections indicating greater temperature variability and more frequent heat extremes, pregnant women may face changing exposures during early pregnancy [50]. We found that moderate cold and heat exposure during early pregnancy is associated with smaller CRLs, suggesting that early gestational development is sensitive to ambient temperature. As environmental conditions shift, these effects could have clinical implications, potentially influencing birth outcomes and long-term health.

## Ethics approval

The Generation R Study (MEC 198.782.2001.31) and Generation R Next Study (MEC 2016–589, NL57828.078.16) were approved by the Medical Ethics Committee of the Erasmus University Medical Center Rotterdam. Written informed consent was obtained from all participants.

## Supplementary Material

dyag060_Supplementary_Data

## Data Availability

This project uses data from the Generation R Next Study (https://generationr.nl/next/). The datasets generated and analyzed during the current study are not publicly available due to legal and ethical regulations, but may be made available upon request to the director of the cohort in accordance with the local, national, and European Union regulations: Director of the Generation R (Next) Study, Vincent Jaddoe (v.jaddoe@erasmusmc.nl). The code to reproduce the analysis will be made available at the open repository “dataverse” of the Consorci de Serveis Universitaris de Catalunya.
