# Supplementary material for: Associations of ambient temperature exposure with embryonic and early fetal development
Source: Int J Epidemiol. 2026 May 24;55(3):dyag060. doi: 10.1093/ije/dyag060 (PMC13198863; doi:10.1093/ije/dyag060)
Supplement: dyag060_Supplementary_Data [file dyag060_supplementary_data.docx]

**SUPPLEMENTARY MATERIAL**

**Associations of Ambient Temperature Exposure
with Embryonic and Early Fetal Development**

| **Figure S1** | Flowchart of the study population | 2 |
| --- | --- | --- |
| **Figure S2** | Distribution of gestational ages at the 2D ultrasound measurements at weeks 8, 10, and 12 of pregnancy | 3 |
| **Methods S1** | Detailed description of the temperature exposure assessment | 4 |
| **Figure S3** | Directed acyclic graph of the temperature and crown-rump length association | 6 |
| **Table S1** | Details of the imputation procedure in the analysis sample | 7 |
| **Table S2** | Population characteristics of participants in observed and expectation-maximization imputed datasets | 8 |
| **Table S3** | Population characteristics of participants included and excluded in the study | 10 |
| **Table S4** | List of variables used as predictors of participation in the covariate balancing propensity score procedure for inverse probability weighting | 12 |
| **Figure S4** | Distribution of inverse probability weights winsorized at 10 for the analysis sample of each study population | 13 |
| **Methods S2** | Description of the crown-rump length growth analysis | 14 |
| **Figure S5** | Distribution of mean weekly ambient temperature by month during weeks 1 to 6 of pregnancy in the replication cohort | 15 |
| **Figure S6** | Cumulative exposure-lag-response associations between weekly ambient temperature exposure for different exposure periods and crown-rump length at 8, 10, and 12 weeks of pregnancy | 16 |
| **Table S5** | Associations between weekly ambient temperature at different lag periods and CRL at 8, 10, and 12 weeks of pregnancy | 17 |
| **Figure S7** | Associations between temperature exposure to cold or heat during each week of pregnancy and CRL at 12 weeks | 21 |
| **Figure S8** | Cumulative associations between weekly ambient temperature at different lag periods and CRL at 8, 10, and 12 weeks of pregnancy, restricted to participants with available CRL measurements for all time points | 22 |
| **Figure S9** | Cumulative association between weekly ambient temperature exposure from pregnancy weeks 1 to 6 and crown-rump length growth from 8 to 12 weeks | 23 |
| **Figure S10** | Cumulative associations between weekly ambient temperature exposure from weeks 1 to 6 (A1) and CRL at 10 weeks extracted from the DLNM with exposure from weeks 1 to 9, and from weeks 1 to 6 (B1) and 1 to 8 (B2) and CRL at 12 weeks extracted from the DLNM with exposure from weeks 1 to 11. | 24 |
| **Figure S11** | Cumulative associations between weekly ambient temperature at different lag periods and CRL at 8, 10, and 12 weeks of pregnancy, DLNM exposure-response relationship modelled with knots at the 10^th^ and 90^th^ percentiles of temperature | 25 |
| **Figure S12** | Cumulative associations between weekly ambient temperature at different lag periods and CRL at 8, 10, and 12 weeks of pregnancy, DLNM lag-response relationship modelled with one knot at the median on the log-scale | 26 |
| **Figure S13** | Cumulative associations between weekly ambient temperature at different lag periods and CRL at 8, 10, and 12 weeks of pregnancy, DLNM lag-response relationship modelled with one knot at the median lag equally spaced | 27 |
| **Figure S14** | Cumulative associations between weekly ambient temperature at different lag periods and CRL at 8, 10, and 12 weeks of pregnancy, DLNM lag-response relationship modelled with two equally spaced knots | 28 |
| **Figure S15** | Cumulative associations between weekly ambient temperature at different lag periods and CRL at 8, 10, and 12 weeks of pregnancy in the replication cohort Generation R | 29 |
| **Figure S16** | Cumulative associations between weekly ambient temperature exposure for different exposure periods and crown-rump length at 12 weeks of pregnancy in the primary cohort Generation R Next and the replication cohort Generation R | 30 |
| **References** |  | 31 |

| 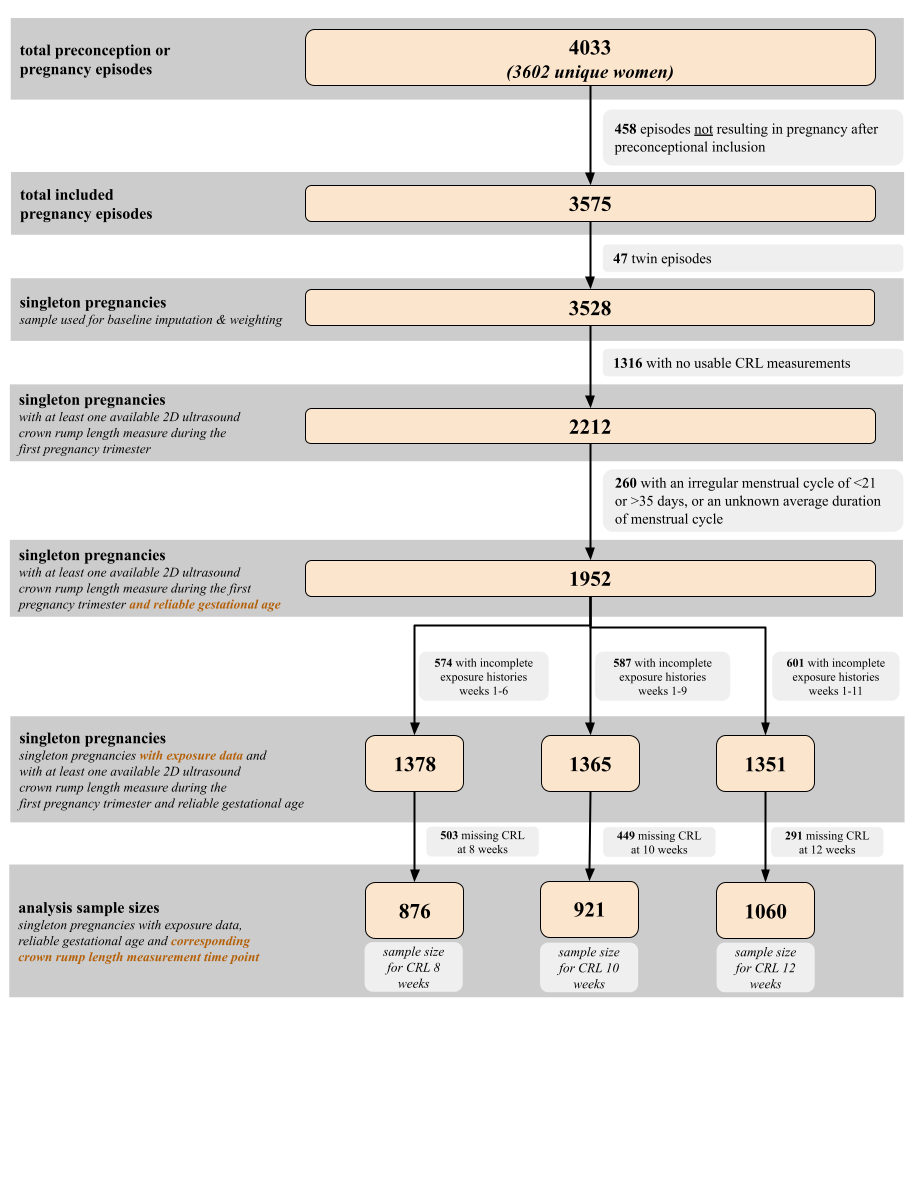 |
| --- |
| **Figure S1. Flowchart of the study population.**  Abbreviations: CRL: crown-rump length. |

| 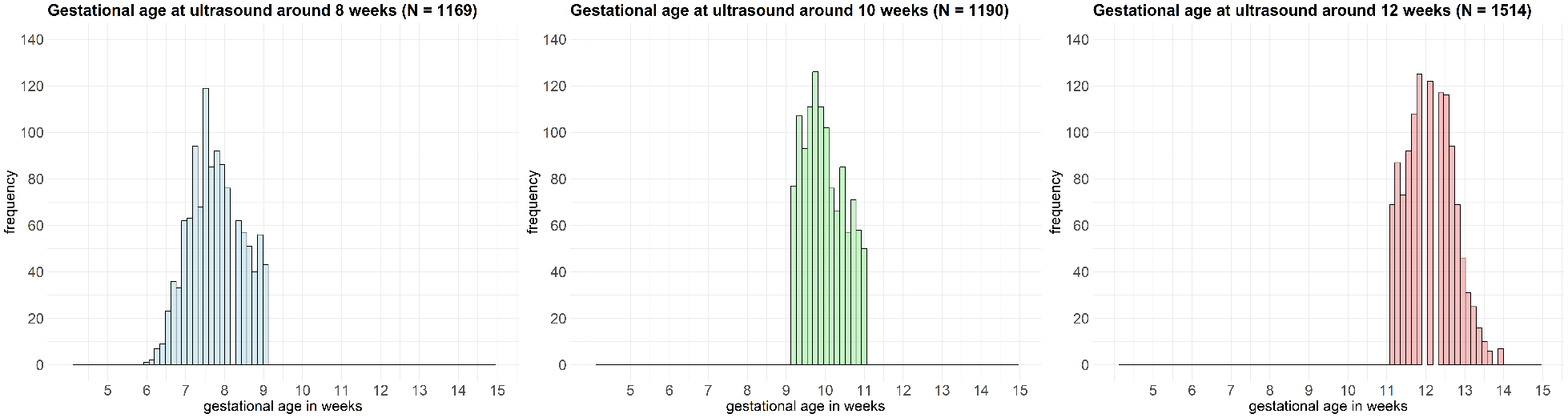 |
| --- |
| **Figure S2. Distribution of gestational ages at the 2D ultrasound measurements at weeks 8, 10, and 12 of pregnancy.** |

**Methods S1. Detailed description of the temperature exposure assessment.**

Accurate assessment of ambient temperature was ensured via the UrbClim^TM^ model (De Ridder et al., 2015). This model uses urban physics coupled with a 3D atmospheric boundary layer module that includes information on the urban structure (e.g., vegetation, land use and cover) to generate climate data at a high temporal (hourly) and spatial (100 x 100 metres) resolution (De Ridder et al., 2015; Golan et al., 2018). UrbClim^TM^ has been successfully validated in various European countries, including Spain (García-Díez et al., 2016; Lauwaet et al., 2015, 2016), and the data has been used in previous projects utilizing Generation R data (Essers et al., 2024, 2025; Granés et al., 2024, 2025). Detailed methodology is described elsewhere (De Ridder et al., 2015; García-Díez et al., 2016; Lauwaet et al., 2015, 2016). The model is developed for domains of 30 x 30 km. For Generation R, two domains (Rotterdam and Den Haag) were placed based on the geographical area where the maximum number of participants lived based on all available geocoded addresses starting from pregnancy (87.5%). Hourly temperature was generated for the years 2001 to 2020. Hourly data was converted into daily data based on the Coordinated Universal Time and for this study estimated at the geocoded address where the participant lived at during pregnancy, accounting for changes in address. The model domains were set up using the European Terrestrial Reference System 1989 (EPSG 3035) as the coordinate system (Annoni et al., 2001). To obtain the most realistic representation for historical climate for the regions, the 2006 European CORINE land cover maps (identifying the spatial distribution of land cover types) was used (Corpernicus, n.d.). Annex A on page 5 shows the conversion of the CORINE classification into the 15 classes of land cover in the UrbClim^TM^ model. The model also uses Copernicus Land Imperviousness datasets (soil sealing maps), the Normalized Difference Vegetation Index from the TERRA satellite platforms MODIS instrument (vegetation cover maps), and data provided by the European Centre for Medium-range Weather Forecasting ERA5 reanalysis (background meteorology) as input data (Corpernicus, n.d.; ECMWF, n.d.; NASA, n.d.).

| ***Annex A. Land use conversion tables from CORINE classes into the fifteen UrbClim^TM^ classes.*** | | | | |
| --- | --- | --- | --- | --- |
| CORINE code | English name | UrbClim code | UrbClim class |  |
| 1 | Continuous urban fabric | 1 | Urban |  |
| 2 | Discont. urban fabric | 2 | Suburban |  |
| 3 | Industrial or commercial units | 3 | Industrial |  |
| 4 | Road/rail networks and ass. land | 3 | Industrial |  |
| 5 | Port areas | 3 | Industrial |  |
| 6 | Airports | 3 | Industrial |  |
| 7 | Mineral extraction sites | 3 | Industrial |  |
| 8 | Dump sites | 3 | Industrial |  |
| 9 | Construction sites | 3 | Industrial |  |
| 10 | Green urban areas | 4 | Urban green |  |
| 11 | Sport and leisure facilities | 4 | Urban green |  |
| 12 | Non-irrigated arable land | 8 | Cropland |  |
| 13 | Permanent irrigated land | 8 | Cropland |  |
| 14 | Rice fields | 8 | Cropland |  |
| 15 | Vineyards | 9 | Shrubland |  |
| 16 | Fruit trees and berry plantations | 10 | Woodland |  |
| 17 | Olive groves | 10 | Woodland |  |
| 18 | Pastures | 7 | Grassland |  |
| 19 | Annual crops w/ permanent crops | 8 | Cropland |  |
| 20 | Complex cultivation patterns | 8 | Cropland |  |
| 21 | Heterogeneous agricultural areas | 8 | Cropland |  |
| 22 | Agro-forestry areas | 10 | Woodland |  |
| 23 | Broad-leaved forest | 11 | Broadleaf trees |  |
| 24 | Coniferous forest | 12 | Needleleaf trees |  |
| 25 | Mixed forest | 11 | Broadleaf trees |  |
| 26 | Natural grasslands | 7 | Grassland |  |
| 27 | Moors and heathland | 10 | Woodland |  |
| 28 | Sclerophyllous vegetation | 9 | Shrubland |  |
| 29 | Transit. woodland-shrub | 9 | Shrubland |  |
| 30 | Beaches, dunes, sand | 6 | Bare soil |  |
| 31 | Bare rocks | 6 | Bare soil |  |
| 32 | Sparsely vegetated areas | 9 | Shrubland |  |
| 33 | Burnt areas | 6 | Bare soil |  |
| 34 | Glaciers & perpetual snow | 5 | Snow/ice |  |
| 35 | Inland marshes | 9 | Shrubland |  |
| 36 | Peat bogs | 9 | Shrubland |  |
| 37 | Salt marshes | 7 | Grassland |  |
| 38 | Salines | 7 | Grassland |  |
| 39 | Intertidal flats | 6 | Bare soil |  |
| 40 | Water courses | 13 | River |  |
| 41 | Water bodies | 14 | Inland water body |  |
| 42 | Coastal lagoons | 15 | Sea |  |
| 43 | Estuaries | 15 | Sea |  |
| 44 | Sea and ocean | 15 | Sea |  |

| 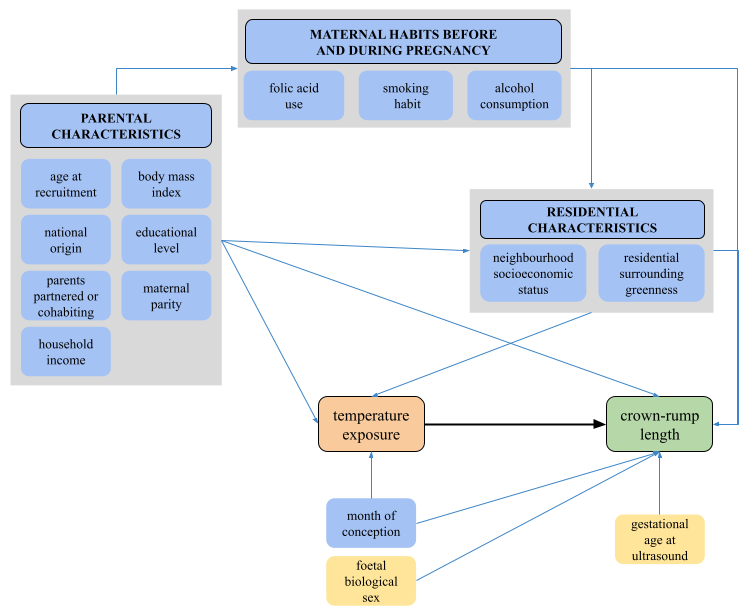 |
| --- |
| **Figure S3. Directed acyclic graph of the temperature and crown-rump length association.**  The node in orange represents the exposure and in green the outcome. Blue nodes and arrows represent potential confounding variables and pathways, yellow nodes represent ancestors of the outcome. |

| **Table S1. Details of the imputation procedure in the analysis samples.** |
| --- |
| **Software used and key setting:** |
| R (version 4.3.2; R Core Team (2023)) – Amelia package v1.8.3 |
| **Number of imputed datasets created:** |
| 1 imputed dataset per sample |
| **Sample size for imputations:** |
| 876 for crown-rump length at 8 weeks  921 for crown-rump length at 10 weeks  1060 for crown-rump length at 12 weeks  1378 for crown-rump length growth between 8 and 12 weeks |
| **Variables with repeated measures averaged into one value for imputation:** |
| Weekly temperature during weeks 1 to 6 of pregnancy for crown-rump length at 8 weeks  Weekly temperature during weeks 1 to 9 of pregnancy for crown-rump length at 10 weeks  Weekly temperature during weeks 1 to 111 of pregnancy for crown-rump length at 12 weeks  Weekly temperature during weeks 1 to 6 of pregnancy for crown-rump length growth |
| **Variables used as predictors for the imputation:** |
| Foetal biological sex  Maternal age at recruitment  Paternal age at recruitment  Maternal pre-pregnancy body mass index  Paternal body mass index at recruitment  Maternal national origin  Paternal national origin  Maternal educational level  Paternal educational level  Parents are partners / cohabiting  Monthly household income  Maternal parity  Maternal smoking habit before and during pregnancy  Maternal alcohol consumption before and during pregnancy  Maternal folic acid use before and during pregnancy  Residential surrounding greenness from conception to ultrasound at 8, 10, or 12 weeks  Residential neighbourhood socioeconomic status from conception to ultrasound at 8, 10, or 12 weeks  Averaged weekly temperature during weeks 1 to 6, 9, or 11 of pregnancy  Crown-rump length at the ultrasounds at 8, 10, or 12 weeks of pregnancy |
| **Diagnostics:** Visual inspection by checking convergence and density plots |
| **References:** (Honaker et al., 2012) |

| **Table S2. Population characteristics of participants in observed and expectation-maximization imputed datasets.** | | | | |
| --- | --- | --- | --- | --- |
|  | | N = 1378 | | % Imputed |
|  | | Observed | Imputed |  |
| *Foetal Characteristics* | |  |  |  |
| Sex (female *vs.* male) | | 47.4 | - | 0.0 |
| Season of conception | |  |  | 0.0 |
|  | Summer | 27.3 | - |  |
|  | Autumn | 27.9 | - |  |
|  | Winter | 19.5 | - |  |
|  | Spring | 25.2 | - |  |
| *Maternal Characteristics* | |  |  |  |
| Age at recruitment (years) | | 31.9 (3.9) | - | 0.0 |
| Body mass index (kg/m^2^) | | 24.3 (4.4) | 24.4 (4.5) | 6.1 |
| National origin | |  |  | 1.9 |
|  | The Netherlands | 66.2 | 65.5 |  |
|  | Suriname / Dutch Caribbean | 10.4 | 10.7 |  |
|  | Other Global South countries^1^ | 13.1 | 13.1 |  |
|  | Other Global North countries^1^ | 10.3 | 10.7 |  |
| Educational level | |  |  | 2.9 |
|  | High | 73.4 | 72.9 |  |
|  | Medium | 22.7 | 22.9 |  |
|  | Low | 3.9 | 4.2 |  |
| Parity | |  |  | 4.1 |
|  | no children | 64.8 | 64.5 |  |
|  | 1 child | 27.2 | 27.3 |  |
|  | 2 or more children | 8.1 | 8.2 |  |
| Smoking habit | |  |  | 10.9 |
|  | Did not smoke | 55.6 | 55.7 |  |
|  | Quit smoking before pregnancy | 31.1 | 30.8 |  |
|  | Smoked during pregnancy | 13.3 | 13.6 |  |
| Alcohol consumption | |  |  | 5.2 |
|  | No consumption <3 months before pregnancy | 19.7 | 20.2 |  |
|  | Consumption <3 months before pregnancy | 63.9 | 63.3 |  |
|  | Consumption during pregnancy | 16.5 | 16.5 |  |
| Folic acid use | |  |  | 5.8 |
|  | Started use prior to pregnancy | 69.8 | 68.7 |  |
|  | Did not use or started during pregnancy | 30.2 | 31.4 |  |
| Values are percentage for categorical and mean (standard deviation) for continuous variables. *P*-value was obtained using the ꭓ^2^-test for categorical variables and Wilcoxon Rank Sum test for continuous variables, and bolded values indicate significance at the 0.05 level. ^1^ Countries categorized as Global South include Turkey, Morocco, Cape Verde, Indonesia, China, countries in Africa, other American and Asian non-western countries; and countries categorized as Global North include Germany, Yugoslavia, Poland, other American and Asian non-western countries, other European and all Oceania countries. ^2^ exposure calculated from conception date until the date of the 8-week ultrasound. | | | | |

| **Table S2, continued. Population characteristics of participants in observed and expectation-maximization imputed datasets.** | | | | |
| --- | --- | --- | --- | --- |
|  | | N = 1370 | | % Imputed |
|  | | Observed | Imputed |  |
| *Paternal Characteristics* | |  |  |  |
| Age at recruitment (years) | | 33.9 (5.1) | 34.0 (5.2) | 11.5 |
| Body mass index (kg/m^2^) | | 25.6 (3.7) | 25.7 (3.7) | 13.9 |
| National origin | |  |  | 5.8 |
|  | The Netherlands | 64.9 | 63.1 |  |
|  | Suriname / Dutch Carribean | 9.6 | 10.2 |  |
|  | Other Global South countries^1^ | 16.6 | 17.3 |  |
|  | Other Global North countries^1^ | 9.0 | 9.4 |  |
| Educational level | |  |  | 5.6 |
|  | High | 63.9 | 62.4 |  |
|  | Medium | 28.5 | 29.2 |  |
|  | Low | 7.6 | 8.3 |  |
| *Residential Characteristics* | | |  |  |
| Residential surrounding greenness^2^ | | 0.4 (0.1) | 0.4 (0.1) | 17.7 |
| Neighbourhood socioeconomic status^2^ | | -0.1 (0.1) | -0.1 (0.1) | 1.7 |
| Partner/co-habiting (yes *vs.* no) | | 89.0 | 88.2 | 5.2 |
| Monthly net household income | |  |  | 12.5 |
|  | < €2000 | 9.6 | 10.4 |  |
|  | €2000 – 4000 | 32.1 | 32.0 |  |
|  | €4000 – 6000 | 43.5 | 42.3 |  |
|  | > €6000 | 14.8 | 15.2 |  |
| Values are percentage for categorical and mean (standard deviation) for continuous variables. *P*-value was obtained using the ꭓ^2^-test for categorical variables and Wilcoxon Rank Sum test for continuous variables, and bolded values indicate significance at the 0.05 level. ^1^ Countries categorized as Global South include Turkey, Morocco, Cape Verde, Indonesia, China, countries in Africa, other American and Asian non-western countries; and countries categorized as Global North include Germany, Yugoslavia, Poland, other American and Asian non-western countries, other European and all Oceania countries. ^2^ exposure calculated from conception date until the date of the 7-week ultrasound. | | | | |

| **Table S3. Population characteristics of participants included and excluded in the study.** | | | | |
| --- | --- | --- | --- | --- |
|  | | Included  (N = 1378) | Not Included  (N = 2150) | *P-value* |
| *Foetal Characteristics* | |  |  |  |
| Sex (female *vs.* male) | | 47.4 | 467.0 | 0.943 |
| Season of conception | |  |  | **0.001** |
|  | Summer | 27.3 | 23.9 |  |
|  | Autumn | 27.9 | 26.6 |  |
|  | Winter | 19.5 | 30.3 |  |
|  | Spring | 25.2 | 19.1 |  |
| *Maternal Characteristics* | |  |  |  |
| Age at recruitment (years) | | 31.9 (3.9) | 31.4 (5.0) | 0.218 |
| Body mass index (kg/m^2^) | | 24.3 (4.4) | 24.4 (4.6) | 0.921 |
| National origin | |  |  | **0.031** |
|  | The Netherlands | 66.2 | 56.5 |  |
|  | Suriname / Dutch Caribbean | 10.4 | 12.7 |  |
|  | Other Global South countries^1^ | 13.1 | 18.8 |  |
|  | Other Global North countries^1^ | 10.3 | 12.0 |  |
| Educational level | |  |  | 0.056 |
|  | High | 73.4 | 66.7 |  |
|  | Medium | 22.7 | 26.3 |  |
|  | Low | 3.9 | 7.0 |  |
| Parity | |  |  | 0.337 |
|  | no children | 64.8 | 61.3 |  |
|  | 1 child | 27.2 | 27.7 |  |
|  | 2 or more children | 8.1 | 101.0 |  |
| Smoking habit | |  |  | 0.146 |
|  | Did not smoke | 55.6 | 53.1 |  |
|  | Quit smoking before pregnancy | 31.1 | 28.4 |  |
|  | Smoked during pregnancy | 13.3 | 18.5 |  |
| Alcohol consumption | |  |  | 0.250 |
|  | No consumption <3 months before pregnancy | 19.7 | 23.1 |  |
|  | Consumption <3 months before pregnancy | 63.9 | 63.8 |  |
|  | Consumption during pregnancy | 16.5 | 13.1 |  |
| Folic acid use | |  |  | **0.007** |
|  | Started use prior to pregnancy | 69.8 | 60.8 |  |
|  | Did not use or started during pregnancy | 30.2 | 39.2 |  |
| Values are percentage for categorical and mean (standard deviation) for continuous variables. *P*-value was obtained using the ꭓ^2^-test for categorical variables and Wilcoxon Rank Sum test for continuous variables, and bolded values indicate significance at the 0.05 level. ^1^ Countries categorized as Global South include Turkey, Morocco, Cape Verde, Indonesia, China, countries in Africa, other American and Asian non-western countries; and countries categorized as Global North include Germany, Yugoslavia, Poland, other American and Asian non-western countries, other European and all Oceania countries. ^2^ exposure calculated from conception date until the date of the 7-week ultrasound. | | | | |

| **Table S3, continued. Population characteristics of participants included and excluded in the study.** | | | | |
| --- | --- | --- | --- | --- |
|  | | Included  (N = 1378) | Not Included  (N = 2150) | *P-value* |
| *Paternal Characteristics* | |  |  |  |
| Age at recruitment (years) | | 33.9 (5.1) | 33.8 (6.0) | 0.781 |
| Body mass index (kg/m^2^) | | 25.6 (3.7) | 25.7 (4.0) | 0.639 |
| National origin | |  |  | 0.180 |
|  | The Netherlands | 64.9 | 57.8 |  |
|  | Suriname / Dutch Carribean | 9.6 | 13.4 |  |
|  | Other Global South countries^1^ | 16.6 | 18.5 |  |
|  | Other Global North countries^1^ | 9.0 | 10.3 |  |
| Educational level | |  |  | 0.211 |
|  | High | 63.9 | 58.6 |  |
|  | Medium | 28.5 | 30.8 |  |
|  | Low | 7.6 | 10.6 |  |
| *Residential Characteristics* | | |  |  |
| Residential surrounding greenness^2^ | | 0.4 (0.1) | 0.3 (0.1) | 0.859 |
| Neighbourhood socioeconomic status^2^ | | -0.1 (0.1) | -0.1 (0.1) | 0.237 |
| Partner/co-habiting (yes *vs.* no) | | 89.0 | 86.3 | 0.261 |
| Monthly net household income | |  |  | 0.17 |
|  | < €2000 | 9.6 | 15.5 |  |
|  | €2000 – 4000 | 32.1 | 31.7 |  |
|  | €4000 – 6000 | 43.5 | 39.3 |  |
|  | > €6000 | 14.8 | 13.5 |  |
| Values are percentage for categorical and mean (standard deviation) for continuous variables. *P*-value was obtained using the ꭓ^2^-test for categorical variables and Wilcoxon Rank Sum test for continuous variables, and bolded values indicate significance at the 0.05 level. ^1^ Countries categorized as Global South include Turkey, Morocco, Cape Verde, Indonesia, China, countries in Africa, other American and Asian non-western countries; and countries categorized as Global North include Germany, Yugoslavia, Poland, other American and Asian non-western countries, other European and all Oceania countries. ^2^ exposure calculated from conception date until the date of the 7-week ultrasound. | | | | |

| **Table S4. List of variables used as predictors of participation in the covariate balancing propensity score procedure for inverse probability weighting.** | |
| --- | --- |
| Foetal biological sex |  |
| Maternal age at recruitment |  |
| Paternal age at recruitment |  |
| Maternal national origin |  |
| Paternal national origin |  |
| Maternal educational level during pregnancy |  |
| Paternal educational level during pregnancy |  |
| Parents partnered / cohabiting |  |
| Household monthly income during pregnancy |  |
| Maternal pre-pregnancy body mass index |  |
| Paternal body mass index at enrolment |  |
| Maternal parity at pregnancy |  |
| Neighbourhood socioeconomic status during pregnancy |  |
| Surrounding household greenness during pregnancy |  |
| Maternal alcohol consumption before and during pregnancy |  |
| Maternal smoking habit before and during pregnancy |  |
| Maternal folic acid use before and during pregnancy |  |

| **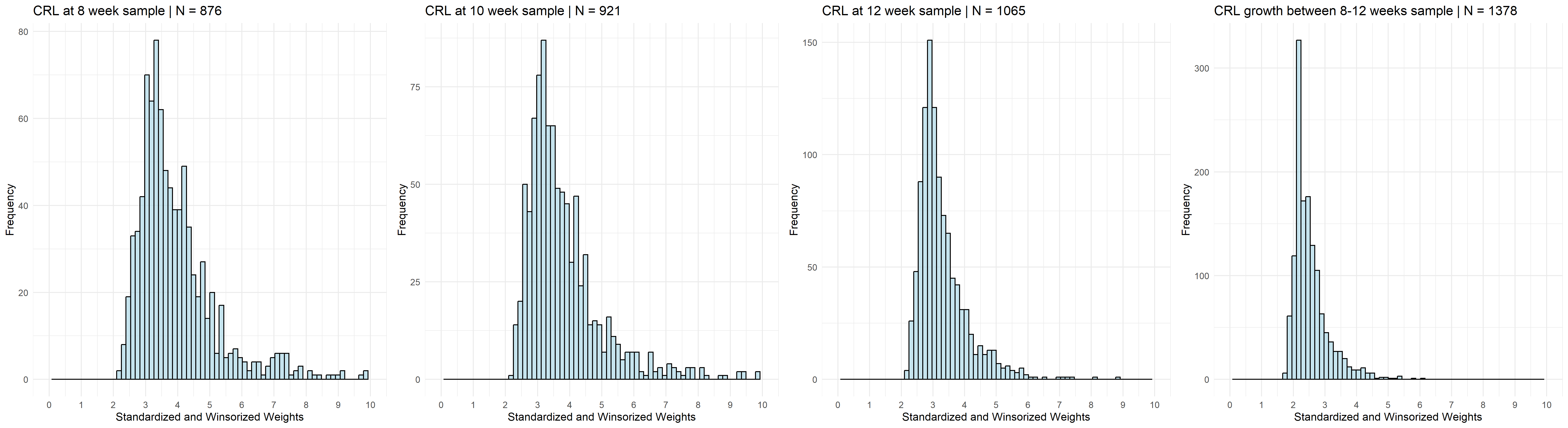**  **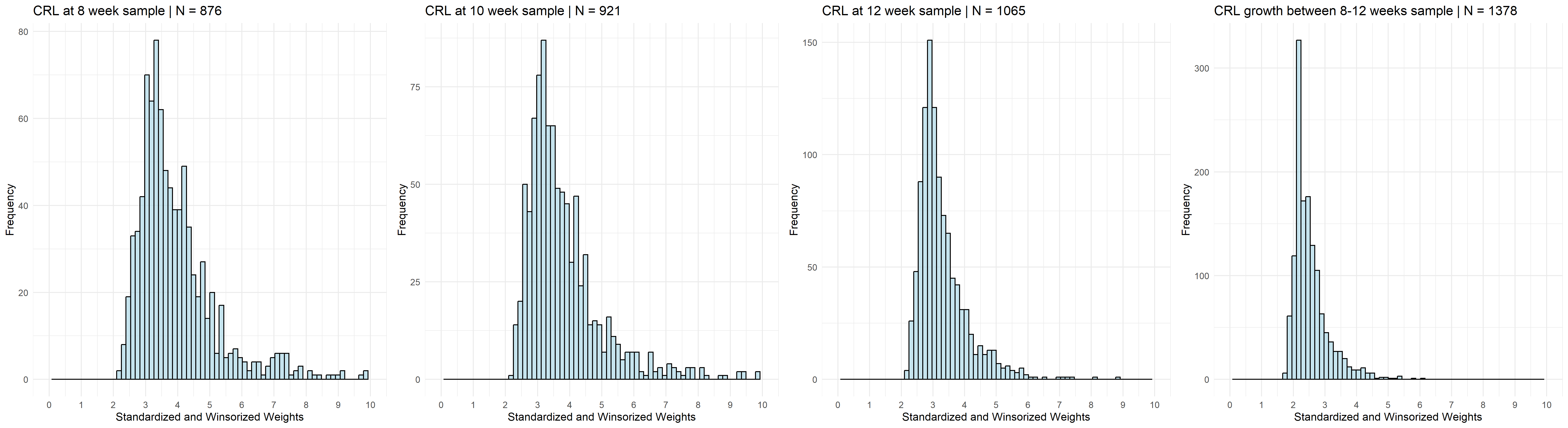** |
| --- |
| **Figure S4. Distribution of inverse probability weights winsorized at 10 for the analysis sample of each study population.**  Abbreviations: CRL: crown-rump length. |

**Methods S2. Description of the crown-rump length growth analysis.**

We evaluated crown-rump length growth using the serial crown-rump length measurements taken at 8, 10, and 12 weeks of gestation, using the change in length as the growth outcome. First, we visually evaluated the slope of crown-rump length and gestational age to confirm linearity (figure below). Then, we estimated participant-specific changes in crown-rump length through fitting a linear mixed model with a fixed intercept and random slope for gestational age. We used gestational age at each ultrasound as a predictor in the model. We explored the inclusion of foetal sex and its interaction with gestational age to account for potential sex-specific growth differences. Including these terms did not improve model fit (AIC comparison) or meaningfully alter individual growth slope estimates (correlation between slopes: r = 0.99). As such, they were not retained in the final model to preserve parsimony. We estimated the crown-rump length growth slope per participant in millimetres per week. We also calculated the inverse variance of the crown-rump length growth slope estimation to use as a weight in analyses using growth as an outcome. In doing this, we improved certainty by assigning a greater weight to those participants that have narrower confidence intervals in the crown-rump length growth slope estimation. Finally, we evaluated the association between weekly ambient temperature exposure during pregnancy weeks 1 to 6 and CRL growth by applying distributed lag non-linear models. Model parameterizations were identical to those of the main analysis.


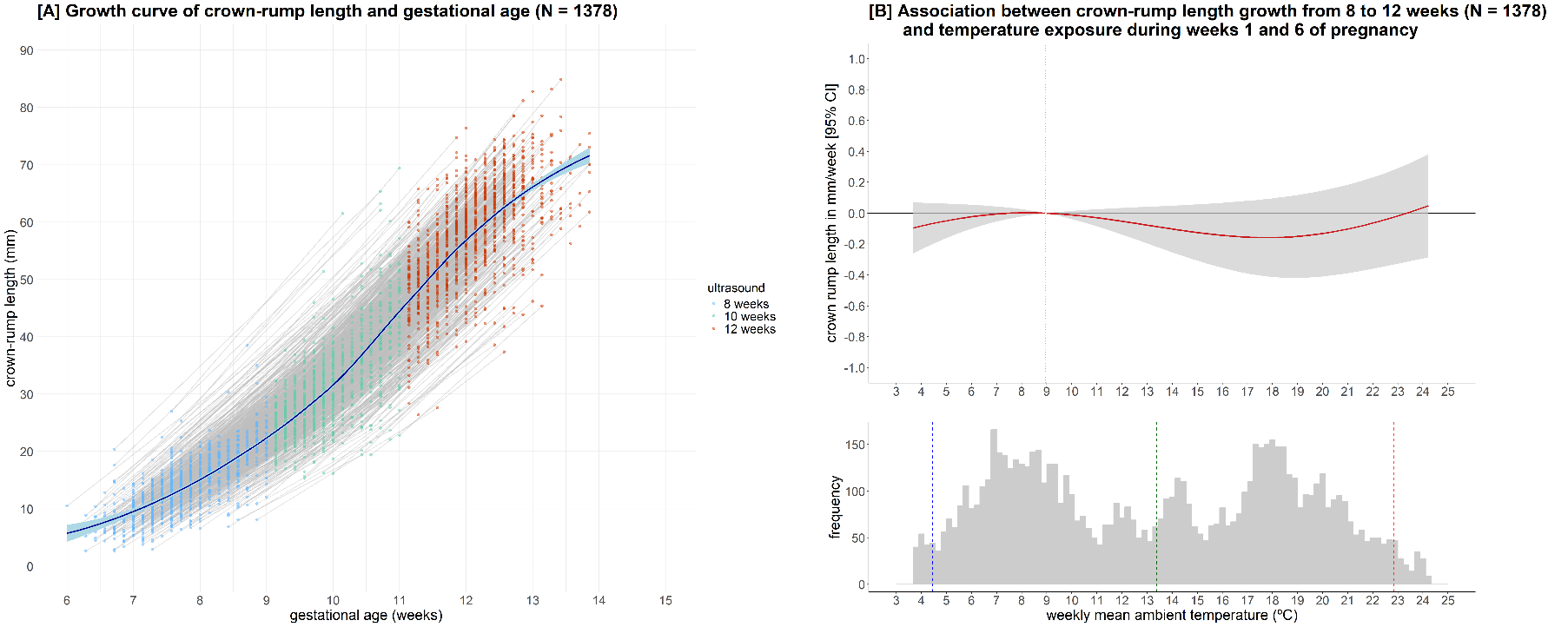


| **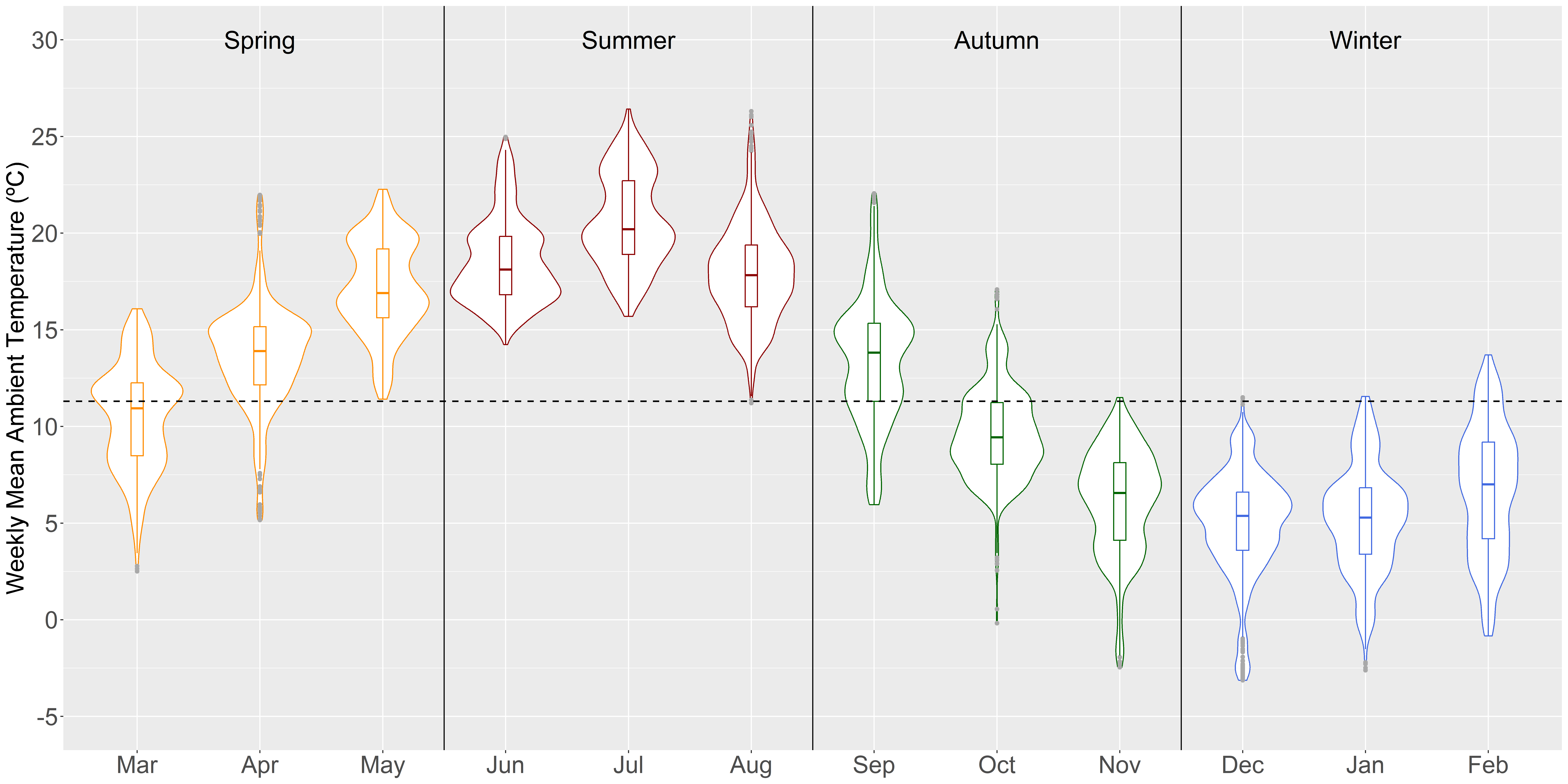** |
| --- |
| **Figure S5. Distribution of mean weekly ambient temperature by month during weeks 1 to 6 of pregnancy in the replication cohort**  Sample size in the replication cohort Generation R is 1520 (participants with at least one crown-rump length measure at the 8, 10, or 12-week ultrasound and temperature exposure for at least weeks 1 to 6 of pregnancy). Temperature exposure is for the years 2001 to 2005. The width of the violin plots indicates the density of temperature observations at that ºC value. The boxplots within the violin plots show the median and interquartile range, with the upper and lower boundaries indicating the maximum and minimum temperature values, respectively. The dashed horizontal line indicates the overall mean temperature at 11.3ºC. |

| **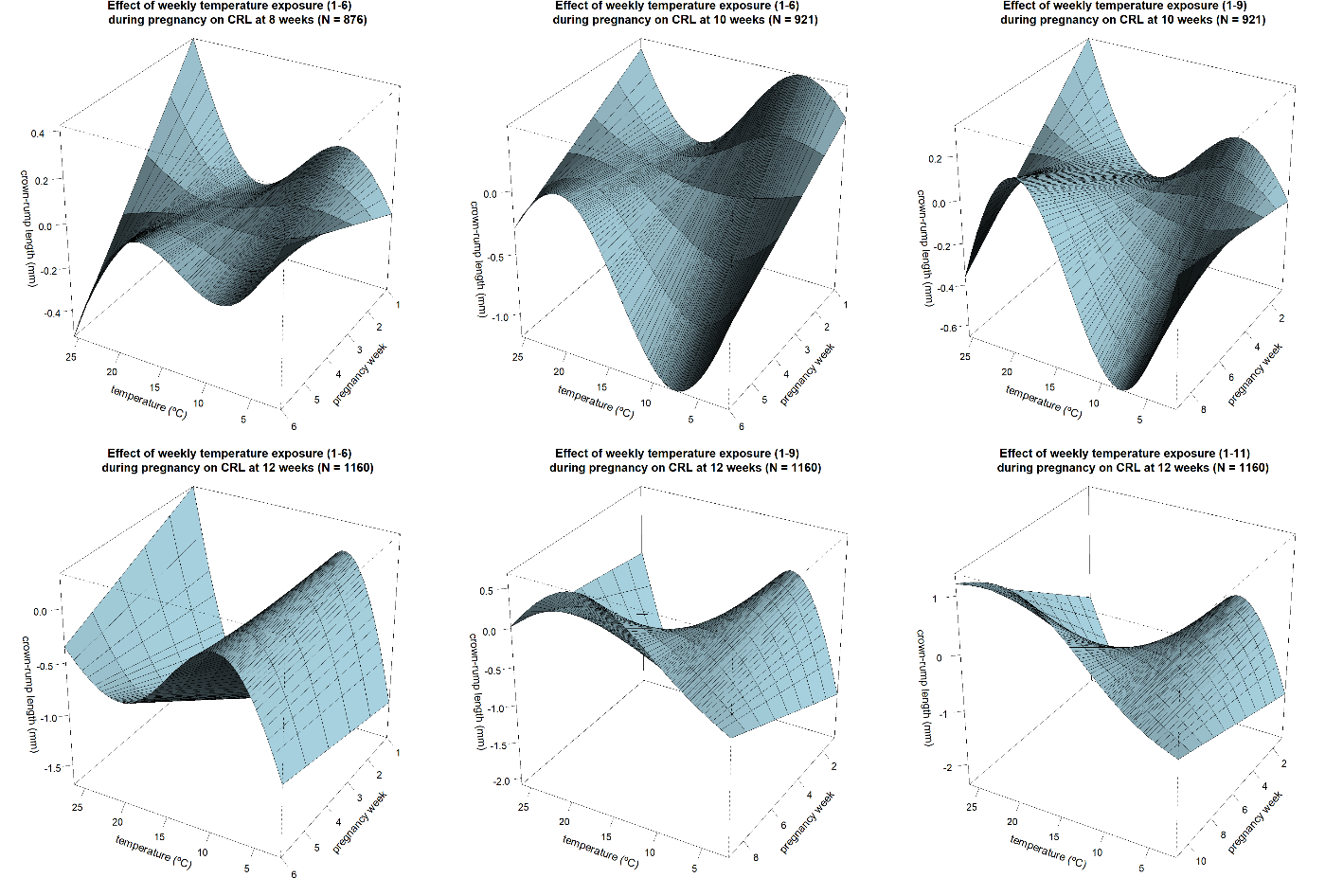** |
| --- |
| **Figure S6. Cumulative exposure-lag-response associations between weekly ambient temperature exposure for different exposure periods and crown-rump length at 8, 10, and 12 weeks of pregnancy.**  The x-axes depict the temperature distribution (in ºC), the y-axes depict the coefficients of crown-rump length (in mm), and the z-axes depict the lag period (in pregnancy weeks). Coefficients are estimated as the change in crown-rump length in millimeters at each temperature during each pregnancy week respective to the outcome-specific reference temperature. Distributed lag non-linear models were adjusted for fetal biological sex; parental age at recruitment, national origin, educational level, and body mass index; parents partnered or co-habiting, monthly net household income, maternal parity, alcohol consumption, smoking habit, folic acid use, and month of last menstrual period; residential surrounding greenness, neighborhood socioeconomic status, and gestational age at the ultrasound. |

| **Table S5. Cumulative associations between weekly ambient temperature exposure for different exposure periods and crown-rump length at 8, 10, and 12 weeks of pregnancy.** | | | | | | | | | | | | | | |
| --- | --- | --- | --- | --- | --- | --- | --- | --- | --- | --- | --- | --- | --- | --- |
|  | crown-rump length at 8 weeks (N = 876) | | | |  | crown-rump length at 10 weeks (N = 921) | | | | | | | | |
|  | *six-week exposure period* | | | |  | *six-week exposure period* | | | |  | *nine-week exposure period* | | | |
| **Perc.** | **Temp. (ºC)** | **Coef.** | **95% CI** | |  | **Temp. (ºC)** | **Coef.** | **95% CI** | |  | **Temp. (ºC)** | **Coef.** | **95% CI** | |
| 1 | 1.8 | 0.10 | -1.04 | 1.24 |  | 1.8 | -0.78 | -6.74 | 5.17 |  | 1.9 | -1.08 | -8.34 | 6.17 |
| 2 | 2.7 | 0.06 | -0.63 | 0.75 |  | 2.9 | -1.15 | -6.79 | 4.49 |  | 3.0 | -1.60 | -8.39 | 5.19 |
| 3 | 3.4 | 0.04 | -0.33 | 0.40 |  | 3.5 | -1.33 | -6.85 | 4.19 |  | 3.4 | -1.79 | -8.43 | 4.86 |
| 4 | 3.8 | 0.02 | -0.16 | 0.19 |  | 3.9 | -1.46 | -6.91 | 3.99 |  | 3.8 | -1.97 | -8.48 | 4.54 |
| 5 | 4.3 | Ref. | Ref. | Ref. |  | 4.2 | -1.55 | -6.95 | 3.86 |  | 4.3 | -2.10 | -8.53 | 4.33 |
| 6 | 4.5 | -0.01 | -0.13 | 0.10 |  | 4.6 | -1.62 | -6.99 | 3.75 |  | 4.6 | -2.23 | -8.58 | 4.13 |
| 7 | 4.9 | -0.03 | -0.28 | 0.23 |  | 4.9 | -1.70 | -7.04 | 3.63 |  | 4.9 | -2.31 | -8.62 | 4.00 |
| 8 | 5.1 | -0.04 | -0.37 | 0.29 |  | 5.1 | -1.76 | -7.07 | 3.56 |  | 5.1 | -2.38 | -8.65 | 3.89 |
| 9 | 5.4 | -0.05 | -0.46 | 0.37 |  | 5.4 | -1.81 | -7.11 | 3.48 |  | 5.4 | -2.45 | -8.68 | 3.78 |
| 10 | 5.6 | -0.06 | -0.55 | 0.43 |  | 5.6 | -1.86 | -7.13 | 3.42 |  | 5.6 | -2.50 | -8.70 | 3.70 |
| 11 | 5.8 | -0.06 | -0.61 | 0.48 |  | 5.8 | -1.89 | -7.15 | 3.37 |  | 5.8 | -2.54 | -8.72 | 3.64 |
| 12 | 6.0 | -0.07 | -0.67 | 0.52 |  | 5.9 | -1.92 | -7.17 | 3.33 |  | 5.9 | -2.58 | -8.73 | 3.57 |
| 13 | 6.2 | -0.08 | -0.73 | 0.57 |  | 6.1 | -1.95 | -7.19 | 3.29 |  | 6.1 | -2.62 | -8.75 | 3.51 |
| 14 | 6.4 | -0.09 | -0.80 | 0.62 |  | 6.3 | -1.98 | -7.20 | 3.24 |  | 6.3 | -2.66 | -8.76 | 3.44 |
| 15 | 6.5 | -0.10 | -0.84 | 0.65 |  | 6.5 | -2.01 | -7.22 | 3.20 |  | 6.5 | -2.68 | -8.77 | 3.40 |
| 16 | 6.8 | -0.10 | -0.91 | 0.70 |  | 6.7 | -2.04 | -7.23 | 3.15 |  | 6.7 | -2.72 | -8.77 | 3.34 |
| 17 | 6.8 | -0.11 | -0.93 | 0.72 |  | 6.8 | -2.05 | -7.23 | 3.13 |  | 6.8 | -2.73 | -8.78 | 3.32 |
| 18 | 6.9 | -0.11 | -0.96 | 0.74 |  | 6.9 | -2.06 | -7.23 | 3.11 |  | 6.9 | -2.74 | -8.78 | 3.29 |
| 19 | 7.1 | -0.12 | -1.00 | 0.76 |  | 7.0 | -2.08 | -7.23 | 3.08 |  | 7.0 | -2.76 | -8.77 | 3.26 |
| 20 | 7.2 | -0.12 | -1.03 | 0.78 |  | 7.2 | -2.09 | -7.23 | 3.05 |  | 7.2 | -2.77 | -8.77 | 3.23 |
| 21 | 7.3 | -0.13 | -1.07 | 0.81 |  | 7.3 | -2.10 | -7.23 | 3.03 |  | 7.3 | -2.78 | -8.77 | 3.20 |
| 22 | 7.4 | -0.14 | -1.10 | 0.83 |  | 7.4 | -2.11 | -7.23 | 3.01 |  | 7.4 | -2.79 | -8.76 | 3.18 |
| 23 | 7.6 | -0.14 | -1.12 | 0.84 |  | 7.5 | -2.12 | -7.23 | 2.98 |  | 7.5 | -2.80 | -8.75 | 3.15 |
| 24 | 7.7 | -0.15 | -1.16 | 0.87 |  | 7.6 | -2.14 | -7.22 | 2.95 |  | 7.6 | -2.81 | -8.74 | 3.13 |
| 25 | 7.8 | -0.15 | -1.19 | 0.88 |  | 7.8 | -2.14 | -7.21 | 2.92 |  | 7.8 | -2.81 | -8.73 | 3.10 |
| 26 | 7.9 | -0.16 | -1.22 | 0.90 |  | 7.9 | -2.15 | -7.20 | 2.90 |  | 7.9 | -2.82 | -8.71 | 3.08 |
| 27 | 8.0 | -0.16 | -1.25 | 0.92 |  | 8.0 | -2.16 | -7.19 | 2.87 |  | 8.0 | -2.82 | -8.69 | 3.05 |
| 28 | 8.2 | -0.17 | -1.27 | 0.94 |  | 8.2 | -2.16 | -7.17 | 2.84 |  | 8.1 | -2.82 | -8.67 | 3.03 |
| 29 | 8.3 | -0.17 | -1.30 | 0.95 |  | 8.3 | -2.17 | -7.15 | 2.82 |  | 8.3 | -2.82 | -8.65 | 3.01 |
| 30 | 8.4 | -0.18 | -1.33 | 0.97 |  | 8.4 | -2.17 | -7.13 | 2.79 |  | 8.4 | -2.82 | -8.63 | 2.99 |
| 31 | 8.5 | -0.19 | -1.36 | 0.98 |  | 8.5 | -2.17 | -7.11 | 2.77 |  | 8.5 | -2.81 | -8.60 | 2.98 |
| 32 | 8.7 | -0.19 | -1.38 | 1.00 |  | 8.6 | -2.17 | -7.09 | 2.75 |  | 8.6 | -2.81 | -8.58 | 2.97 |
| 33 | 8.8 | -0.20 | -1.40 | 1.01 |  | 8.8 | -2.17 | -7.07 | 2.72 |  | 8.7 | -2.80 | -8.55 | 2.95 |
| 34 | 9.0 | -0.20 | -1.43 | 1.03 |  | 8.9 | -2.17 | -7.03 | 2.69 |  | 8.9 | -2.79 | -8.52 | 2.94 |
| 35 | 9.1 | -0.21 | -1.46 | 1.04 |  | 9.1 | -2.17 | -7.00 | 2.66 |  | 9.0 | -2.78 | -8.47 | 2.92 |
| 36 | 9.2 | -0.22 | -1.50 | 1.06 |  | 9.2 | -2.17 | -6.97 | 2.64 |  | 9.2 | -2.77 | -8.44 | 2.91 |
| 37 | 9.4 | -0.22 | -1.52 | 1.07 |  | 9.3 | -2.16 | -6.94 | 2.62 |  | 9.3 | -2.76 | -8.41 | 2.90 |
| 38 | 9.5 | -0.23 | -1.55 | 1.09 |  | 9.5 | -2.15 | -6.90 | 2.59 |  | 9.4 | -2.74 | -8.36 | 2.89 |
| 39 | 9.7 | -0.24 | -1.59 | 1.11 |  | 9.7 | -2.15 | -6.85 | 2.56 |  | 9.6 | -2.72 | -8.31 | 2.88 |
| 40 | 9.8 | -0.25 | -1.62 | 1.13 |  | 9.8 | -2.14 | -6.82 | 2.54 |  | 9.8 | -2.70 | -8.27 | 2.87 |
| 41 | 10.0 | -0.25 | -1.65 | 1.14 |  | 9.9 | -2.13 | -6.78 | 2.52 |  | 9.9 | -2.68 | -8.22 | 2.86 |
| 42 | 10.2 | -0.26 | -1.68 | 1.16 |  | 10.1 | -2.11 | -6.71 | 2.48 |  | 10.0 | -2.65 | -8.15 | 2.86 |
| 43 | 10.4 | -0.27 | -1.74 | 1.19 |  | 10.4 | -2.09 | -6.63 | 2.44 |  | 10.3 | -2.60 | -8.06 | 2.85 |
| 44 | 10.6 | -0.28 | -1.78 | 1.22 |  | 10.7 | -2.07 | -6.54 | 2.40 |  | 10.6 | -2.56 | -7.97 | 2.85 |
| 45 | 10.9 | -0.29 | -1.84 | 1.25 |  | 11.0 | -2.03 | -6.42 | 2.36 |  | 10.9 | -2.49 | -7.84 | 2.86 |
| 46 | 11.3 | -0.31 | -1.92 | 1.30 |  | 11.4 | -1.98 | -6.28 | 2.31 |  | 11.3 | -2.40 | -7.68 | 2.88 |
| 47 | 11.6 | -0.32 | -1.99 | 1.34 |  | 11.7 | -1.94 | -6.17 | 2.28 |  | 11.7 | -2.31 | -7.53 | 2.90 |
| 48 | 11.8 | -0.34 | -2.05 | 1.38 |  | 11.9 | -1.91 | -6.09 | 2.26 |  | 11.9 | -2.26 | -7.45 | 2.92 |
| 49 | 12.0 | -0.35 | -2.11 | 1.41 |  | 12.1 | -1.88 | -6.01 | 2.24 |  | 12.1 | -2.20 | -7.35 | 2.95 |
| 50 | 12.3 | -0.36 | -2.16 | 1.45 |  | 12.3 | -1.85 | -5.93 | 2.23 |  | 12.4 | -2.13 | -7.24 | 2.98 |
| Coefficients were obtained from distributed lag non-linear models and are estimated as the millimetre change in crown-rump length at each temperature of the lag period-specific temperature distribution between percentile 1 and 99, respective to the reference temperature (Ref.). Bold values indicate associations that are significant at the 0.05 level and grey shaded values indicate associations that survived correction for multiple testing (P-value < 0.025). Distributed lag non-linear models were adjusted for foetal biological sex, parental age at intake, parental national origin, parental educational level, parents partnered or co-habiting, monthly net household income, maternal parity, maternal alcohol consumption, maternal smoking habit, maternal folic acid use, parental body mass index, residential surrounding greenness, neighbourhood socioeconomic status, month of conception, and gestational age at the ultrasound. Abbreviations: CI, confidence interval; Coef., coefficient; Perc., percentile; Ref., reference; Temp., temperature. | | | | | | | | | | | | | | |
| **Table S5, continued. Cumulative associations between weekly ambient temperature exposure for different exposure periods and crown-rump length at 8, 10, and 12 weeks of pregnancy.** | | | | | | | | | | | | | | |
|  | crown-rump length at 8 weeks (N = 876) | | | |  | crown-rump length at 10 weeks (N = 921) | | | | | | | | |
|  | *six-week exposure period* | | | |  | *six-week exposure period* | | | |  | *nine-week exposure period* | | | |
| **Perc.** | **Temp. (ºC)** | **Coef.** | **95% CI** | |  | **Temp. (ºC)** | **Coef.** | **95% CI** | |  | **Temp. (ºC)** | **Coef.** | **95% CI** | |
| 51 | 12.5 | -0.37 | -2.21 | 1.48 |  | 12.6 | -1.80 | -5.80 | 2.21 |  | 12.8 | -2.03 | -7.09 | 3.02 |
| 52 | 13.2 | -0.39 | -2.34 | 1.57 |  | 13.2 | -1.69 | -5.56 | 2.18 |  | 13.3 | -1.85 | -6.82 | 3.12 |
| 53 | 13.6 | -0.41 | -2.47 | 1.65 |  | 13.6 | -1.62 | -5.43 | 2.18 |  | 13.7 | -1.74 | -6.67 | 3.19 |
| 54 | 13.9 | -0.42 | -2.55 | 1.71 |  | 13.8 | -1.58 | -5.34 | 2.17 |  | 13.9 | -1.67 | -6.56 | 3.23 |
| 55 | 14.1 | -0.43 | -2.61 | 1.76 |  | 14.1 | -1.53 | -5.24 | 2.17 |  | 14.1 | -1.59 | -6.45 | 3.28 |
| 56 | 14.3 | -0.43 | -2.66 | 1.79 |  | 14.2 | -1.50 | -5.17 | 2.17 |  | 14.3 | -1.54 | -6.38 | 3.31 |
| 57 | 14.4 | -0.44 | -2.69 | 1.82 |  | 14.4 | -1.47 | -5.11 | 2.18 |  | 14.5 | -1.48 | -6.30 | 3.35 |
| 58 | 14.6 | -0.44 | -2.74 | 1.85 |  | 14.5 | -1.43 | -5.05 | 2.18 |  | 14.6 | -1.42 | -6.23 | 3.38 |
| 59 | 14.8 | -0.45 | -2.79 | 1.89 |  | 14.7 | -1.40 | -4.97 | 2.18 |  | 14.9 | -1.35 | -6.12 | 3.42 |
| 60 | 15.1 | -0.46 | -2.87 | 1.95 |  | 15.0 | -1.33 | -4.85 | 2.19 |  | 15.2 | -1.24 | -5.96 | 3.48 |
| 61 | 15.5 | -0.47 | -2.97 | 2.02 |  | 15.4 | -1.24 | -4.68 | 2.20 |  | 15.6 | -1.12 | -5.78 | 3.54 |
| 62 | 15.8 | -0.48 | -3.02 | 2.07 |  | 15.7 | -1.18 | -4.56 | 2.20 |  | 15.9 | -1.03 | -5.64 | 3.57 |
| 63 | 16.1 | -0.48 | -3.08 | 2.11 |  | 16.0 | -1.13 | -4.46 | 2.21 |  | 16.1 | -0.96 | -5.53 | 3.60 |
| 64 | 16.3 | -0.49 | -3.13 | 2.15 |  | 16.3 | -1.08 | -4.36 | 2.21 |  | 16.4 | -0.89 | -5.40 | 3.62 |
| 65 | 16.6 | -0.49 | -3.18 | 2.19 |  | 16.5 | -1.02 | -4.25 | 2.20 |  | 16.6 | -0.83 | -5.28 | 3.62 |
| 66 | 16.8 | -0.50 | -3.22 | 2.22 |  | 16.8 | -0.97 | -4.15 | 2.20 |  | 16.8 | -0.77 | -5.17 | 3.63 |
| 67 | 17.0 | -0.50 | -3.25 | 2.25 |  | 17.0 | -0.92 | -4.04 | 2.19 |  | 17.0 | -0.72 | -5.07 | 3.62 |
| 68 | 17.2 | -0.50 | -3.28 | 2.27 |  | 17.2 | -0.88 | -3.95 | 2.18 |  | 17.2 | -0.67 | -4.96 | 3.61 |
| 69 | 17.3 | -0.51 | -3.31 | 2.30 |  | 17.3 | -0.86 | -3.89 | 2.17 |  | 17.4 | -0.63 | -4.87 | 3.60 |
| 70 | 17.5 | -0.51 | -3.33 | 2.32 |  | 17.5 | -0.83 | -3.81 | 2.16 |  | 17.5 | -0.60 | -4.78 | 3.59 |
| 71 | 17.6 | -0.51 | -3.35 | 2.33 |  | 17.6 | -0.80 | -3.74 | 2.14 |  | 17.6 | -0.56 | -4.68 | 3.56 |
| 72 | 17.8 | -0.51 | -3.37 | 2.35 |  | 17.7 | -0.77 | -3.67 | 2.13 |  | 17.8 | -0.52 | -4.59 | 3.54 |
| 73 | 17.9 | -0.51 | -3.38 | 2.36 |  | 17.9 | -0.75 | -3.60 | 2.11 |  | 17.9 | -0.50 | -4.52 | 3.52 |
| 74 | 18.0 | -0.51 | -3.39 | 2.37 |  | 18.0 | -0.72 | -3.54 | 2.09 |  | 18.0 | -0.47 | -4.44 | 3.49 |
| 75 | 18.1 | -0.51 | -3.41 | 2.38 |  | 18.1 | -0.70 | -3.47 | 2.07 |  | 18.1 | -0.45 | -4.37 | 3.46 |
| 76 | 18.2 | -0.51 | -3.42 | 2.40 |  | 18.2 | -0.68 | -3.40 | 2.05 |  | 18.2 | -0.42 | -4.27 | 3.43 |
| 77 | 18.3 | -0.51 | -3.43 | 2.41 |  | 18.4 | -0.66 | -3.35 | 2.03 |  | 18.4 | -0.40 | -4.19 | 3.39 |
| 78 | 18.5 | -0.51 | -3.44 | 2.42 |  | 18.5 | -0.63 | -3.26 | 2.00 |  | 18.5 | -0.37 | -4.07 | 3.33 |
| 79 | 18.7 | -0.51 | -3.46 | 2.43 |  | 18.6 | -0.60 | -3.15 | 1.96 |  | 18.7 | -0.34 | -3.95 | 3.27 |
| 80 | 18.8 | -0.51 | -3.47 | 2.45 |  | 18.8 | -0.57 | -3.06 | 1.92 |  | 18.8 | -0.31 | -3.82 | 3.20 |
| 81 | 19.0 | -0.51 | -3.48 | 2.46 |  | 19.0 | -0.54 | -2.97 | 1.88 |  | 19.0 | -0.28 | -3.69 | 3.13 |
| 82 | 19.3 | -0.51 | -3.50 | 2.48 |  | 19.2 | -0.50 | -2.80 | 1.80 |  | 19.3 | -0.25 | -3.50 | 3.01 |
| 83 | 19.5 | -0.51 | -3.51 | 2.50 |  | 19.4 | -0.46 | -2.64 | 1.72 |  | 19.5 | -0.21 | -3.31 | 2.88 |
| 84 | 19.6 | -0.50 | -3.52 | 2.51 |  | 19.6 | -0.42 | -2.49 | 1.65 |  | 19.6 | -0.19 | -3.14 | 2.77 |
| 85 | 19.8 | -0.50 | -3.53 | 2.53 |  | 19.8 | -0.39 | -2.37 | 1.58 |  | 19.8 | -0.17 | -2.99 | 2.66 |
| 86 | 20.0 | -0.50 | -3.54 | 2.54 |  | 19.9 | -0.37 | -2.24 | 1.51 |  | 20.0 | -0.15 | -2.83 | 2.54 |
| 87 | 20.2 | -0.50 | -3.55 | 2.55 |  | 20.1 | -0.34 | -2.12 | 1.44 |  | 20.1 | -0.13 | -2.66 | 2.41 |
| 88 | 20.4 | -0.49 | -3.56 | 2.57 |  | 20.3 | -0.31 | -1.95 | 1.34 |  | 20.4 | -0.11 | -2.46 | 2.25 |
| 89 | 20.6 | -0.49 | -3.57 | 2.59 |  | 20.5 | -0.28 | -1.78 | 1.23 |  | 20.6 | -0.09 | -2.23 | 2.06 |
| 90 | 20.8 | -0.49 | -3.59 | 2.61 |  | 20.7 | -0.24 | -1.60 | 1.12 |  | 20.8 | -0.07 | -2.02 | 1.88 |
| 91 | 21.0 | -0.48 | -3.60 | 2.64 |  | 20.9 | -0.21 | -1.42 | 1.00 |  | 21.0 | -0.05 | -1.80 | 1.69 |
| 92 | 21.4 | -0.48 | -3.63 | 2.68 |  | 21.2 | -0.17 | -1.18 | 0.84 |  | 21.3 | -0.04 | -1.46 | 1.39 |
| 93 | 21.8 | -0.46 | -3.67 | 2.74 |  | 21.6 | -0.11 | -0.78 | 0.56 |  | 21.7 | -0.02 | -0.95 | 0.92 |
| 94 | 22.2 | -0.45 | -3.71 | 2.81 |  | 22.0 | -0.05 | -0.37 | 0.27 |  | 22.1 | 0.00 | -0.45 | 0.44 |
| 95 | 22.6 | -0.44 | -3.76 | 2.88 |  | 22.4 | Ref. | Ref. | Ref. |  | 22.5 | Ref. | Ref. | Ref. |
| 96 | 23.0 | -0.43 | -3.84 | 2.99 |  | 22.8 | 0.06 | -0.34 | 0.45 |  | 23.0 | 0.00 | -0.63 | 0.63 |
| 97 | 23.9 | -0.40 | -4.02 | 3.23 |  | 23.7 | 0.15 | -0.98 | 1.28 |  | 23.7 | -0.02 | -1.67 | 1.63 |
| 98 | 24.6 | -0.37 | -4.23 | 3.50 |  | 24.4 | 0.24 | -1.67 | 2.14 |  | 24.4 | -0.05 | -2.73 | 2.62 |
| 99 | 25.5 | -0.32 | -4.60 | 3.96 |  | 25.5 | 0.35 | -2.77 | 3.48 |  | 25.4 | -0.12 | -4.36 | 4.12 |
|  |  |  |  |  |  |  |  |  |  |  |  |  |  |  |
| Coefficients were obtained from distributed lag non-linear models and are estimated as the millimetre change in crown-rump length at each temperature of the lag period-specific temperature distribution between percentile 1 and 99, respective to the reference temperature (Ref.). Bold values indicate associations that are significant at the 0.05 level and grey shaded values indicate associations that survived correction for multiple testing (P-value < 0.025). Distributed lag non-linear models were adjusted for foetal biological sex, parental age at intake, parental national origin, parental educational level, parents partnered or co-habiting, monthly net household income, maternal parity, maternal alcohol consumption, maternal smoking habit, maternal folic acid use, parental body mass index, residential surrounding greenness, neighbourhood socioeconomic status, month of conception, and gestational age at the ultrasound. Abbreviations: CI, confidence interval; Coef., coefficient; Perc., percentile; Ref., reference; Temp., temperature. | | | | | | | | | | | | | | |
| **Table S5, continued. Cumulative associations between weekly ambient temperature exposure for different exposure periods and crown-rump length at 8, 10, and 12 weeks of pregnancy.** | | | | | | | | | | | | | | |
|  | crown-rump length at 12 weeks (N = 1160) | | | | | | | | | | | | | |
|  | *six-week exposure period* | | | |  | *nine-week exposure period* | | | |  | *eleven-week exposure period* | | | |
| **Perc.** | **Temp. (ºC)** | **Coef.** | **95% CI** | |  | **Temp. (ºC)** | **Coef.** | **95% CI** | |  | **Temp. (ºC)** | **Coef.** | **95% CI** | |
| 1 | **2.1** | **-6.98** | **-11.99** | **-1.97** |  | **2.1** | **-9.01** | **-14.77** | **-3.25** |  | **2.0** | **-11.90** | **-18.23** | **-5.57** |
| 2 | **3.1** | **-5.05** | **-8.97** | **-1.12** |  | **3.1** | **-6.78** | **-11.32** | **-2.24** |  | **3.1** | **-8.90** | **-13.83** | **-3.96** |
| 3 | **3.7** | **-4.12** | **-7.51** | **-0.72** |  | **3.6** | **-5.73** | **-9.69** | **-1.77** |  | **3.6** | **-7.60** | **-11.94** | **-3.26** |
| 4 | **4.1** | **-3.46** | **-6.47** | **-0.45** |  | **4.1** | **-4.86** | **-8.34** | **-1.38** |  | **4.1** | **-6.44** | **-10.24** | **-2.64** |
| 5 | **4.5** | **-2.97** | **-5.69** | **-0.25** |  | **4.4** | **-4.26** | **-7.40** | **-1.11** |  | **4.4** | **-5.67** | **-9.12** | **-2.23** |
| 6 | **4.9** | **-2.34** | **-4.69** | **0.00** |  | **4.9** | **-3.51** | **-6.23** | **-0.79** |  | **4.9** | **-4.68** | **-7.66** | **-1.71** |
| 7 | 5.2 | -1.98 | -4.09 | 0.14 |  | **5.2** | **-3.07** | **-5.54** | **-0.60** |  | **5.2** | **-4.09** | **-6.79** | **-1.40** |
| 8 | 5.5 | -1.65 | -3.56 | 0.26 |  | **5.4** | **-2.66** | **-4.88** | **-0.43** |  | **5.5** | **-3.59** | **-6.05** | **-1.14** |
| 9 | 5.7 | -1.43 | -3.20 | 0.33 |  | **5.7** | **-2.35** | **-4.40** | **-0.30** |  | **5.7** | **-3.20** | **-5.46** | **-0.94** |
| 10 | 5.9 | -1.24 | -2.88 | 0.40 |  | **5.9** | **-2.11** | **-4.02** | **-0.21** |  | **5.9** | **-2.89** | **-5.00** | **-0.78** |
| 11 | 6.1 | -1.05 | -2.55 | 0.45 |  | **6.1** | **-1.80** | **-3.51** | **-0.08** |  | **6.1** | **-2.50** | **-4.41** | **-0.59** |
| 12 | 6.4 | -0.85 | -2.20 | 0.51 |  | 6.3 | -1.54 | -3.09 | 0.01 |  | **6.3** | **-2.18** | **-3.92** | **-0.43** |
| 13 | 6.5 | -0.71 | -1.96 | 0.54 |  | 6.5 | -1.36 | -2.79 | 0.08 |  | **6.5** | **-1.93** | **-3.54** | **-0.32** |
| 14 | 6.7 | -0.56 | -1.68 | 0.57 |  | 6.7 | -1.15 | -2.44 | 0.14 |  | **6.7** | **-1.66** | **-3.12** | **-0.20** |
| 15 | 6.8 | -0.48 | -1.55 | 0.58 |  | 6.8 | -1.03 | -2.24 | 0.18 |  | **6.8** | **-1.50** | **-2.88** | **-0.13** |
| 16 | 7.0 | -0.41 | -1.41 | 0.59 |  | 6.9 | -0.92 | -2.05 | 0.21 |  | **7.0** | **-1.36** | **-2.66** | **-0.07** |
| 17 | 7.1 | -0.34 | -1.27 | 0.59 |  | 7.1 | -0.80 | -1.84 | 0.24 |  | **7.1** | **-1.20** | **-2.40** | **-0.01** |
| 18 | 7.2 | -0.27 | -1.13 | 0.59 |  | 7.2 | -0.70 | -1.67 | 0.26 |  | 7.2 | -1.07 | -2.18 | 0.04 |
| 19 | 7.4 | -0.20 | -1.00 | 0.59 |  | 7.3 | -0.61 | -1.51 | 0.28 |  | 7.3 | -0.95 | -1.99 | 0.08 |
| 20 | 7.5 | -0.15 | -0.87 | 0.58 |  | 7.5 | -0.52 | -1.34 | 0.29 |  | 7.5 | -0.83 | -1.78 | 0.12 |
| 21 | 7.7 | -0.09 | -0.73 | 0.56 |  | 7.6 | -0.42 | -1.14 | 0.30 |  | 7.6 | -0.69 | -1.54 | 0.16 |
| 22 | 7.8 | -0.04 | -0.61 | 0.53 |  | 7.8 | -0.34 | -0.98 | 0.30 |  | 7.7 | -0.58 | -1.35 | 0.19 |
| 23 | 7.9 | -0.01 | -0.51 | 0.50 |  | 7.9 | -0.27 | -0.83 | 0.29 |  | 7.9 | -0.47 | -1.15 | 0.20 |
| 24 | 8.1 | 0.02 | -0.41 | 0.46 |  | 8.1 | -0.20 | -0.67 | 0.27 |  | 8.0 | -0.38 | -0.98 | 0.21 |
| 25 | 8.3 | 0.04 | -0.32 | 0.40 |  | 8.2 | -0.15 | -0.54 | 0.25 |  | 8.2 | -0.30 | -0.81 | 0.21 |
| 26 | 8.4 | 0.05 | -0.23 | 0.34 |  | 8.3 | -0.10 | -0.41 | 0.22 |  | 8.3 | -0.22 | -0.64 | 0.20 |
| 27 | 8.5 | 0.05 | -0.17 | 0.28 |  | 8.5 | -0.06 | -0.29 | 0.17 |  | 8.4 | -0.16 | -0.49 | 0.18 |
| 28 | 8.7 | 0.05 | -0.11 | 0.20 |  | 8.6 | -0.04 | -0.20 | 0.13 |  | 8.6 | -0.11 | -0.37 | 0.15 |
| 29 | 8.9 | 0.03 | -0.05 | 0.10 |  | 8.8 | -0.01 | -0.10 | 0.07 |  | 8.7 | -0.07 | -0.27 | 0.12 |
| 30 | 9.0 | Ref. | Ref. | Ref. |  | 8.9 | Ref. | Ref. | Ref. |  | 8.8 | -0.03 | -0.13 | 0.07 |
| 31 | 9.2 | -0.03 | -0.10 | 0.04 |  | 9.1 | 0.00 | -0.09 | 0.10 |  | 9.0 | Ref. | Ref. | Ref. |
| 32 | 9.3 | -0.06 | -0.19 | 0.06 |  | 9.2 | 0.00 | -0.17 | 0.17 |  | 9.1 | 0.02 | -0.08 | 0.12 |
| 33 | 9.4 | -0.11 | -0.30 | 0.08 |  | 9.3 | -0.01 | -0.25 | 0.23 |  | 9.3 | 0.03 | -0.14 | 0.20 |
| 34 | 9.6 | -0.18 | -0.46 | 0.10 |  | 9.5 | -0.03 | -0.38 | 0.31 |  | 9.4 | 0.03 | -0.24 | 0.30 |
| 35 | 9.8 | -0.25 | -0.60 | 0.10 |  | 9.7 | -0.07 | -0.52 | 0.38 |  | 9.6 | 0.02 | -0.37 | 0.40 |
| 36 | 9.9 | -0.32 | -0.74 | 0.11 |  | 9.8 | -0.11 | -0.64 | 0.42 |  | 9.8 | 0.00 | -0.50 | 0.49 |
| 37 | 10.1 | -0.43 | -0.96 | 0.10 |  | 10.0 | -0.15 | -0.75 | 0.46 |  | 9.9 | -0.03 | -0.61 | 0.55 |
| 38 | 10.4 | -0.59 | -1.26 | 0.07 |  | 10.2 | -0.23 | -0.98 | 0.52 |  | 10.1 | -0.07 | -0.77 | 0.62 |
| 39 | 10.7 | -0.78 | -1.60 | 0.03 |  | 10.5 | -0.36 | -1.29 | 0.57 |  | 10.4 | -0.16 | -1.04 | 0.71 |
| 40 | **11.1** | **-1.10** | **-2.15** | **-0.06** |  | 10.9 | -0.53 | -1.67 | 0.61 |  | 10.6 | -0.28 | -1.34 | 0.78 |
| 41 | **11.4** | **-1.33** | **-2.53** | **-0.14** |  | 11.3 | -0.78 | -2.19 | 0.62 |  | 11.0 | -0.48 | -1.81 | 0.85 |
| 42 | **11.7** | **-1.56** | **-2.91** | **-0.22** |  | 11.5 | -0.96 | -2.54 | 0.62 |  | 11.4 | -0.69 | -2.26 | 0.88 |
| 43 | **11.9** | **-1.74** | **-3.20** | **-0.29** |  | 11.8 | -1.13 | -2.87 | 0.60 |  | 11.6 | -0.86 | -2.61 | 0.89 |
| 44 | **12.1** | **-1.98** | **-3.58** | **-0.38** |  | 12.0 | -1.30 | -3.18 | 0.58 |  | 11.8 | -1.01 | -2.90 | 0.89 |
| 45 | **12.4** | **-2.19** | **-3.91** | **-0.46** |  | 12.3 | -1.52 | -3.58 | 0.54 |  | 12.0 | -1.19 | -3.25 | 0.87 |
| 46 | **12.6** | **-2.44** | **-4.32** | **-0.57** |  | 12.5 | -1.72 | -3.94 | 0.51 |  | 12.3 | -1.42 | -3.68 | 0.85 |
| 47 | **13.0** | **-2.80** | **-4.88** | **-0.72** |  | 12.8 | -1.98 | -4.40 | 0.45 |  | 12.6 | -1.64 | -4.10 | 0.81 |
| 48 | **13.3** | **-3.17** | **-5.46** | **-0.88** |  | 13.1 | -2.26 | -4.91 | 0.38 |  | 12.9 | -1.90 | -4.56 | 0.76 |
| 49 | **13.6** | **-3.45** | **-5.89** | **-1.00** |  | 13.5 | -2.60 | -5.49 | 0.29 |  | 13.2 | -2.20 | -5.10 | 0.70 |
| 50 | **13.8** | **-3.63** | **-6.18** | **-1.08** |  | 13.7 | -2.84 | -5.89 | 0.22 |  | 13.5 | -2.53 | -5.68 | 0.62 |
| Coefficients were obtained from distributed lag non-linear models and are estimated as the millimetre change in crown-rump length at each temperature of the lag period-specific temperature distribution between percentile 1 and 99, respective to the reference temperature (Ref.). Bold values indicate associations that are significant at the 0.05 level and grey shaded values indicate associations that survived correction for multiple testing (P-value < 0.025). Distributed lag non-linear models were adjusted for foetal biological sex, parental age at intake, parental national origin, parental educational level, parents partnered or co-habiting, monthly net household income, maternal parity, maternal alcohol consumption, maternal smoking habit, maternal folic acid use, parental body mass index, residential surrounding greenness, neighbourhood socioeconomic status, month of conception, and gestational age at the ultrasound. Abbreviations: CI, confidence interval; Coef., coefficient; Perc., percentile; Ref., reference; Temp., temperature. | | | | | | | | | | | | | | |
| **Table S5, continued. Cumulative associations between weekly ambient temperature exposure at different lag periods and crown-rump length at 8, 10, and 12 weeks of pregnancy.** | | | | | | | | | | | | | | |
|  | crown-rump length at 12 weeks (N = 1160) | | | | | | | | | | | | | |
|  | *six-week exposure period* | | | |  | *nine-week exposure period* | | | |  | *eleven-week exposure period* | | | |
| **Perc.** | **Temp. (ºC)** | **Coef.** | **95% CI** | |  | **Temp. (ºC)** | **Coef.** | **95% CI** | |  | **Temp. (ºC)** | **Coef.** | **95% CI** | |
| 51 | **14.0** | **-3.86** | **-6.54** | **-1.18** |  | 13.9 | -3.01 | -6.19 | 0.17 |  | 13.8 | -2.74 | -6.05 | 0.56 |
| 52 | **14.2** | **-4.04** | **-6.82** | **-1.26** |  | 14.1 | -3.21 | -6.53 | 0.11 |  | 14.0 | -2.94 | -6.39 | 0.51 |
| 53 | **14.3** | **-4.21** | **-7.08** | **-1.33** |  | 14.3 | -3.36 | -6.78 | 0.07 |  | 14.2 | -3.13 | -6.72 | 0.46 |
| 54 | **14.5** | **-4.38** | **-7.35** | **-1.41** |  | **14.5** | **-3.56** | **-7.12** | **-0.00** |  | 14.3 | -3.27 | -6.96 | 0.41 |
| 55 | **14.7** | **-4.57** | **-7.64** | **-1.49** |  | **14.6** | **-3.71** | **-7.38** | **-0.05** |  | 14.5 | -3.47 | -7.29 | 0.36 |
| 56 | **15.0** | **-4.84** | **-8.07** | **-1.62** |  | **14.9** | **-3.98** | **-7.82** | **-0.14** |  | 14.7 | -3.64 | -7.59 | 0.30 |
| 57 | **15.3** | **-5.19** | **-8.60** | **-1.77** |  | **15.3** | **-4.28** | **-8.32** | **-0.24** |  | 15.0 | -3.96 | -8.12 | 0.21 |
| 58 | **15.6** | **-5.46** | **-9.03** | **-1.88** |  | **15.6** | **-4.56** | **-8.78** | **-0.33** |  | 15.4 | -4.30 | -8.69 | 0.10 |
| 59 | **15.9** | **-5.70** | **-9.41** | **-1.99** |  | **15.9** | **-4.80** | **-9.19** | **-0.42** |  | 15.7 | -4.56 | -9.13 | 0.01 |
| 60 | **16.1** | **-5.88** | **-9.69** | **-2.06** |  | **16.1** | **-5.01** | **-9.53** | **-0.49** |  | **16.0** | **-4.80** | **-9.54** | **-0.07** |
| 61 | **16.4** | **-6.10** | **-10.05** | **-2.15** |  | **16.3** | **-5.19** | **-9.82** | **-0.56** |  | **16.2** | **-4.95** | **-9.78** | **-0.11** |
| 62 | **16.7** | **-6.29** | **-10.35** | **-2.22** |  | **16.6** | **-5.35** | **-10.09** | **-0.61** |  | **16.4** | **-5.13** | **-10.08** | **-0.17** |
| 63 | **16.9** | **-6.44** | **-10.60** | **-2.28** |  | **16.8** | **-5.51** | **-10.35** | **-0.67** |  | **16.7** | **-5.28** | **-10.34** | **-0.22** |
| 64 | **17.0** | **-6.55** | **-10.78** | **-2.32** |  | **17.0** | **-5.63** | **-10.56** | **-0.71** |  | **16.9** | **-5.40** | **-10.55** | **-0.25** |
| 65 | **17.2** | **-6.64** | **-10.92** | **-2.35** |  | **17.1** | **-5.74** | **-10.74** | **-0.75** |  | **17.1** | **-5.51** | **-10.74** | **-0.28** |
| 66 | **17.3** | **-6.69** | **-11.01** | **-2.37** |  | **17.2** | **-5.81** | **-10.85** | **-0.77** |  | **17.2** | **-5.59** | **-10.87** | **-0.30** |
| 67 | **17.4** | **-6.76** | **-11.13** | **-2.39** |  | **17.4** | **-5.88** | **-10.96** | **-0.79** |  | **17.3** | **-5.64** | **-10.97** | **-0.32** |
| 68 | **17.5** | **-6.82** | **-11.23** | **-2.40** |  | **17.5** | **-5.94** | **-11.06** | **-0.82** |  | **17.4** | **-5.70** | **-11.06** | **-0.33** |
| 69 | **17.6** | **-6.87** | **-11.32** | **-2.42** |  | **17.6** | **-5.99** | **-11.16** | **-0.83** |  | **17.6** | **-5.74** | **-11.15** | **-0.34** |
| 70 | **17.8** | **-6.92** | **-11.41** | **-2.43** |  | **17.7** | **-6.05** | **-11.26** | **-0.85** |  | **17.7** | **-5.79** | **-11.23** | **-0.34** |
| 71 | **17.9** | **-6.96** | **-11.49** | **-2.44** |  | **17.8** | **-6.10** | **-11.33** | **-0.86** |  | **17.8** | **-5.83** | **-11.31** | **-0.35** |
| 72 | **18.0** | **-7.01** | **-11.57** | **-2.44** |  | **17.9** | **-6.15** | **-11.42** | **-0.88** |  | **17.9** | **-5.86** | **-11.37** | **-0.35** |
| 73 | **18.1** | **-7.04** | **-11.65** | **-2.44** |  | **18.1** | **-6.19** | **-11.50** | **-0.89** |  | **18.0** | **-5.89** | **-11.43** | **-0.35** |
| 74 | **18.2** | **-7.08** | **-11.71** | **-2.44** |  | **18.2** | **-6.24** | **-11.59** | **-0.90** |  | **18.2** | **-5.91** | **-11.49** | **-0.34** |
| 75 | **18.4** | **-7.11** | **-11.78** | **-2.44** |  | **18.3** | **-6.28** | **-11.66** | **-0.90** |  | **18.3** | **-5.94** | **-11.54** | **-0.33** |
| 76 | **18.5** | **-7.13** | **-11.83** | **-2.43** |  | **18.5** | **-6.32** | **-11.73** | **-0.91** |  | **18.4** | **-5.95** | **-11.58** | **-0.32** |
| 77 | **18.6** | **-7.15** | **-11.88** | **-2.42** |  | **18.6** | **-6.35** | **-11.79** | **-0.91** |  | **18.6** | **-5.95** | **-11.61** | **-0.30** |
| 78 | **18.8** | **-7.17** | **-11.94** | **-2.40** |  | **18.8** | **-6.38** | **-11.85** | **-0.90** |  | **18.8** | **-5.95** | **-11.64** | **-0.27** |
| 79 | **18.9** | **-7.17** | **-11.97** | **-2.37** |  | **18.9** | **-6.39** | **-11.89** | **-0.89** |  | **18.9** | **-5.94** | **-11.65** | **-0.23** |
| 80 | **19.2** | **-7.17** | **-12.01** | **-2.32** |  | **19.1** | **-6.41** | **-11.95** | **-0.87** |  | **19.1** | **-5.91** | **-11.64** | **-0.17** |
| 81 | **19.4** | **-7.15** | **-12.03** | **-2.26** |  | **19.3** | **-6.41** | **-11.98** | **-0.84** |  | **19.3** | **-5.86** | **-11.61** | **-0.11** |
| 82 | **19.6** | **-7.12** | **-12.03** | **-2.20** |  | **19.5** | **-6.40** | **-12.00** | **-0.80** |  | **19.5** | **-5.81** | **-11.58** | **-0.04** |
| 83 | **19.7** | **-7.08** | **-12.02** | **-2.13** |  | **19.7** | **-6.38** | **-12.01** | **-0.76** |  | 19.7 | -5.75 | -11.53 | 0.04 |
| 84 | **19.9** | **-7.03** | **-11.99** | **-2.06** |  | **19.9** | **-6.36** | **-12.00** | **-0.71** |  | 19.8 | -5.68 | -11.47 | 0.12 |
| 85 | **20.1** | **-6.97** | **-11.96** | **-1.98** |  | **20.0** | **-6.33** | **-11.99** | **-0.66** |  | 20.0 | -5.60 | -11.40 | 0.21 |
| 86 | **20.3** | **-6.88** | **-11.90** | **-1.86** |  | **20.3** | **-6.27** | **-11.96** | **-0.57** |  | 20.2 | -5.47 | -11.30 | 0.35 |
| 87 | **20.5** | **-6.79** | **-11.83** | **-1.74** |  | **20.5** | **-6.20** | **-11.92** | **-0.48** |  | 20.4 | -5.35 | -11.19 | 0.49 |
| 88 | **20.7** | **-6.69** | **-11.76** | **-1.62** |  | **20.7** | **-6.13** | **-11.88** | **-0.39** |  | 20.6 | -5.22 | -11.07 | 0.64 |
| 89 | **20.9** | **-6.57** | **-11.66** | **-1.48** |  | **20.9** | **-6.05** | **-11.82** | **-0.27** |  | 20.9 | -5.05 | -10.92 | 0.83 |
| 90 | **21.2** | **-6.41** | **-11.53** | **-1.28** |  | **21.1** | **-5.95** | **-11.76** | **-0.14** |  | 21.1 | -4.88 | -10.78 | 1.02 |
| 91 | **21.5** | **-6.21** | **-11.37** | **-1.05** |  | 21.4 | -5.81 | -11.66 | 0.05 |  | 21.4 | -4.63 | -10.57 | 1.31 |
| 92 | **21.8** | **-5.96** | **-11.16** | **-0.76** |  | 21.7 | -5.60 | -11.53 | 0.33 |  | 21.7 | -4.27 | -10.28 | 1.73 |
| 93 | **22.2** | **-5.61** | **-10.87** | **-0.34** |  | 22.1 | -5.32 | -11.36 | 0.72 |  | 22.1 | -3.82 | -9.93 | 2.28 |
| 94 | 22.6 | -5.19 | -10.53 | 0.15 |  | 22.5 | -5.03 | -11.19 | 1.14 |  | 22.6 | -3.31 | -9.56 | 2.94 |
| 95 | 23.0 | -4.82 | -10.24 | 0.60 |  | 22.9 | -4.69 | -11.01 | 1.64 |  | 23.0 | -2.77 | -9.20 | 3.66 |
| 96 | 23.8 | -3.88 | -9.52 | 1.77 |  | 23.4 | -4.19 | -10.78 | 2.41 |  | 23.5 | -2.09 | -8.79 | 4.62 |
| 97 | 24.4 | -3.02 | -8.91 | 2.88 |  | 24.2 | -3.43 | -10.50 | 3.64 |  | 24.3 | -0.77 | -8.10 | 6.55 |
| 98 | 25.3 | -1.74 | -8.07 | 4.59 |  | 25.2 | -2.25 | -10.17 | 5.67 |  | 25.3 | 0.86 | -7.40 | 9.13 |
| 99 | 26.4 | -0.10 | -7.09 | 6.90 |  | 26.2 | -0.92 | -9.93 | 8.08 |  | 26.2 | 2.70 | -6.77 | 12.17 |
|  |  |  |  |  |  |  |  |  |  |  |  |  |  |  |
| Coefficients were obtained from distributed lag non-linear models and are estimated as the millimetre change in crown-rump length at each temperature of the lag period-specific temperature distribution between percentile 1 and 99, respective to the reference temperature (Ref.). Bold values indicate associations that are significant at the 0.05 level and grey shaded values indicate associations that survived correction for multiple testing (P-value < 0.025). Distributed lag non-linear models were adjusted for foetal biological sex, parental age at intake, parental national origin, parental educational level, parents partnered or co-habiting, monthly net household income, maternal parity, maternal alcohol consumption, maternal smoking habit, maternal folic acid use, parental body mass index, residential surrounding greenness, neighbourhood socioeconomic status, month of conception, and gestational age at the ultrasound. Abbreviations: CI, confidence interval; Coef., coefficient; Perc., percentile; Ref., reference; Temp., temperature. | | | | | | | | | | | | | | |

| **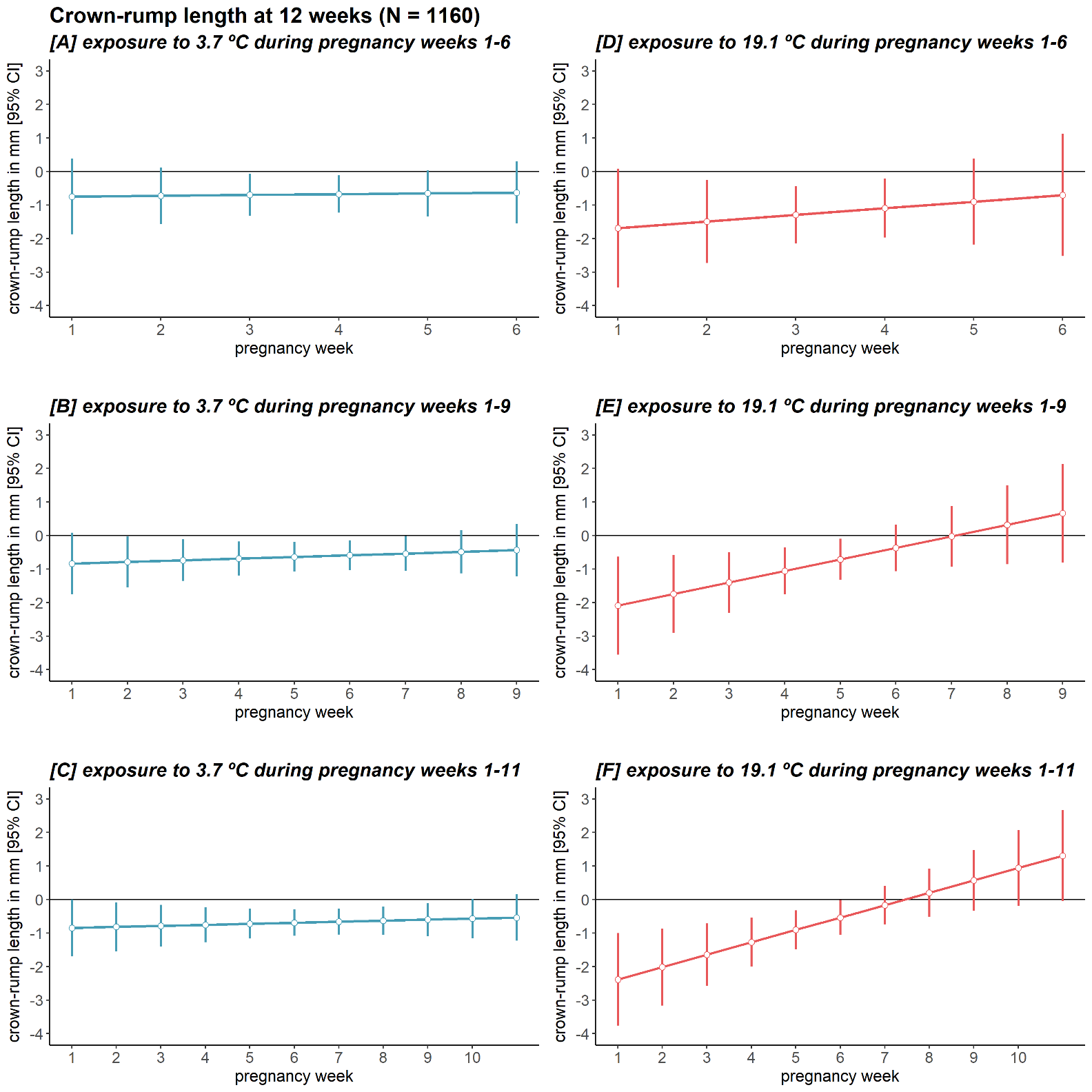** |
| --- |
| **Figure S7. Associations between ambient temperature exposure to cold (blue, 3.7 ºC) or heat (red, 19.1 ºC) during each week of pregnancy and crown-rump length at 12 weeks.**  Dots represent the estimate of the association between the exposure to cold or heat at each pregnancy week and the crown-rump length at 12 weeks with the 95% confidence intervals as vertical lines obtained from distributed lag non-linear models. Blue or red dots and vertical lines indicate exposure to 3.7 and 19.1 ºC, respectively, centered to 9.0 ºC. Distributed lag non-linear models were adjusted for fetal biological sex; parental age at recruitment, national origin, educational level, and body mass index; parents partnered or co-habiting, monthly net household income, maternal parity, alcohol consumption, smoking habit, folic acid use, and month of last menstrual period; residential surrounding greenness, neighborhood socioeconomic status, and gestational age at the ultrasound. Abbreviations: CI, confidence interval. |

| **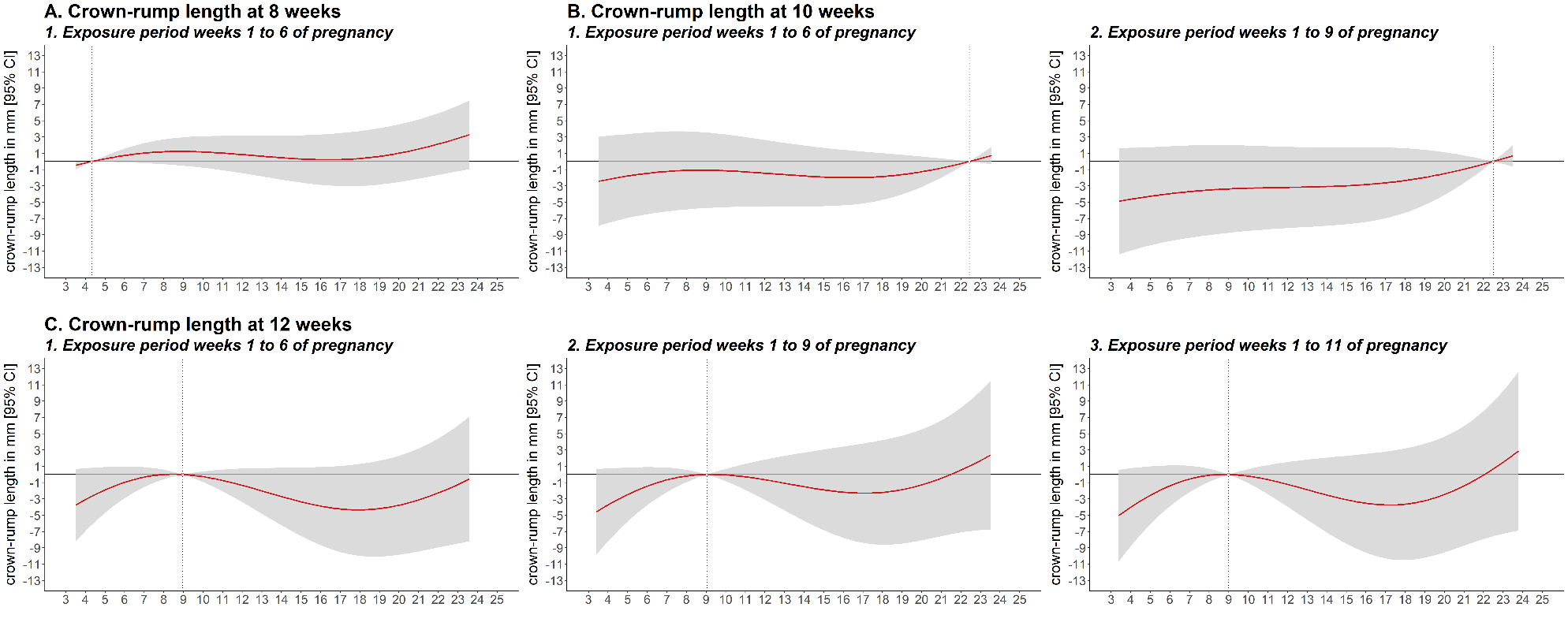** |
| --- |
| **Figure S8. Cumulative associations between weekly ambient temperature exposure for different exposure periods and crown-rump length at 8, 10, and 12 weeks of pregnancy, restricted to participants with available crown-rump length measurements for all time points (N = 495).**  The x-axes depict the 3^rd^ to 97^th^ percentiles of the temperature distribution. Solid red lines represent the associations derived from the distributed lag non-linear models, expressed as beta coefficients of the crown-rump length with their respective 95% confidence intervals in grey. Coefficients are estimated as the change in crown-rump length in millimeters at each temperature respective to the reference temperature (black dotted lines). Distributed lag non-linear models were adjusted for fetal biological sex; parental age at recruitment, national origin, educational level, and body mass index; parents partnered or co-habiting, monthly net household income, maternal parity, alcohol consumption, smoking habit, folic acid use, and month of last menstrual period; residential surrounding greenness, neighborhood socioeconomic status, and gestational age at the ultrasound. Abbreviations: CI, confidence interval. |

| **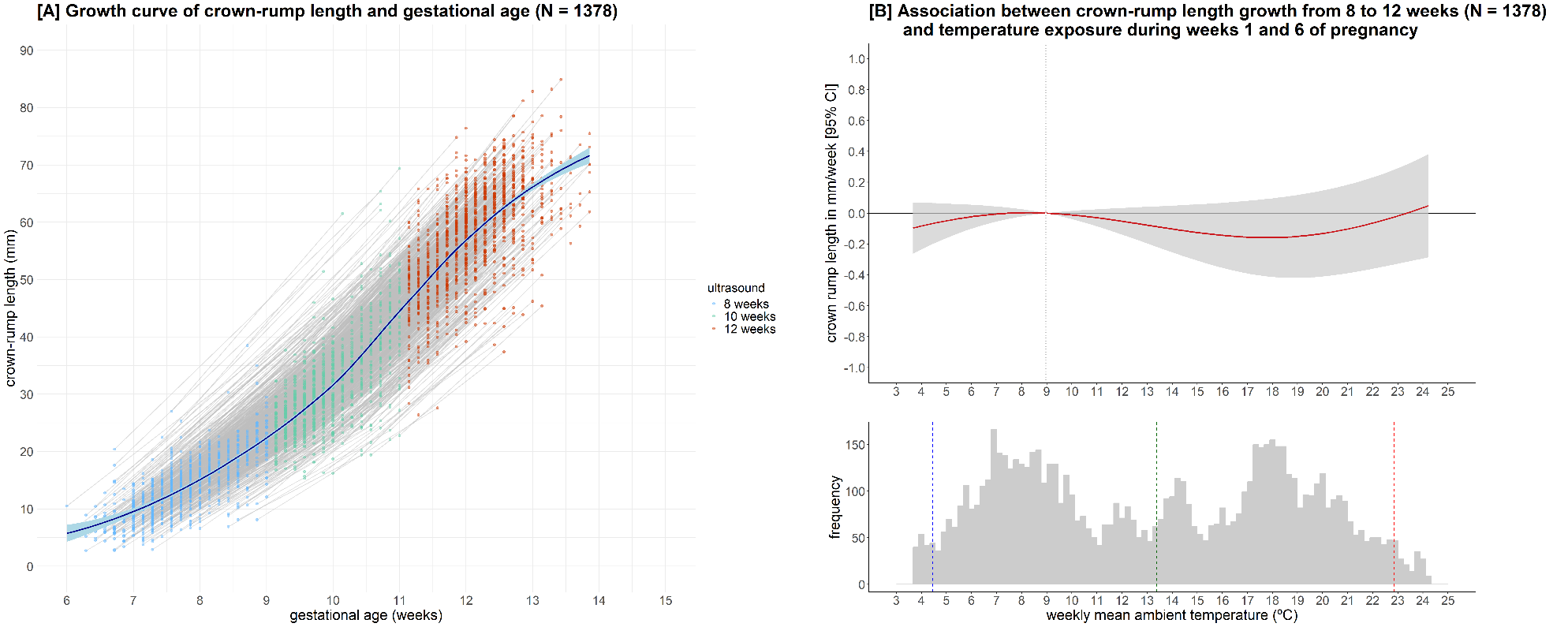** |
| --- |
| **Figure S9. Cumulative association between weekly ambient temperature exposure from pregnancy weeks 1 to 6 and crown-rump length growth from 8 to 12 weeks (N = 1378).**  The x-axes depict the 3^rd^ to 97^th^ percentiles of the temperature distribution. Solid red lines represent the associations derived from the distributed lag non-linear models, expressed as beta coefficients of the crown-rump length with their respective 95% confidence intervals in grey. Coefficients are estimated as the change in crown-rump length in millimeters at each temperature respective to the reference temperature (black dotted lines). Distributed lag non-linear models were adjusted for fetal biological sex; parental age at recruitment, national origin, educational level, and body mass index; parents partnered or co-habiting, monthly net household income, maternal parity, alcohol consumption, smoking habit, folic acid use, and month of last menstrual period; residential surrounding greenness, neighborhood socioeconomic status, and gestational age at the ultrasound. Abbreviations: CI, confidence interval. |

| **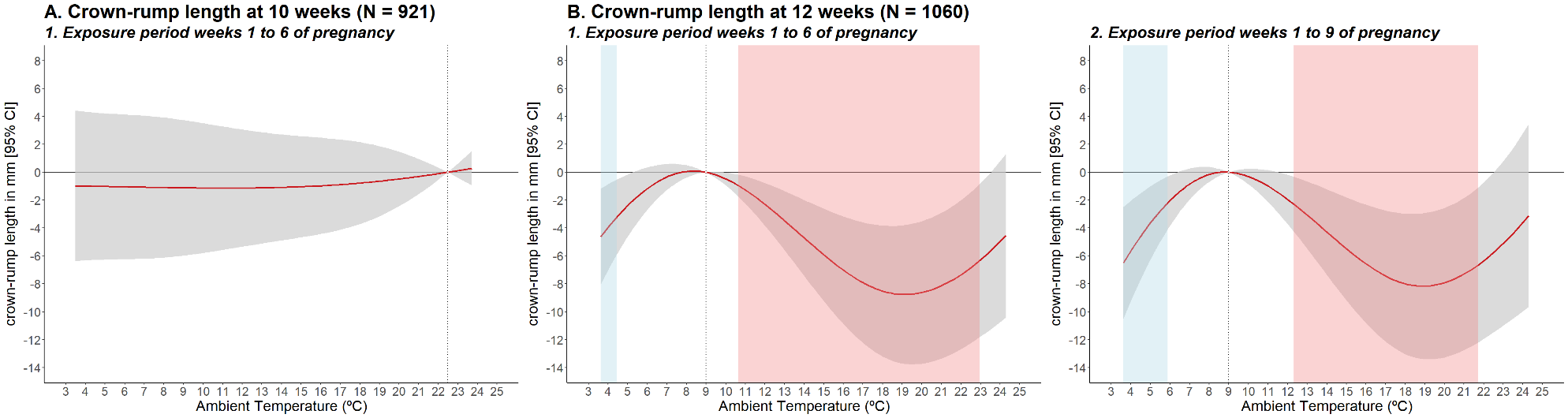** |
| --- |
| **Figure S10. Cumulative associations between weekly ambient temperature exposure from weeks 1 to 6 (A1) and CRL at 10 weeks extracted from the DLNM with exposure from weeks 1 to 9, and from weeks 1 to 6 (B1) and 1 to 9 (B2) and CRL at 12 weeks extracted from the DLNM with exposure from weeks 1 to 11.**  The x-axes depict the 3^rd^ to 97^th^ percentiles of the temperature distribution (**A:** 3.5 to 23.7 ºC, **B:** 3.6 to 24.3 ºC). In the upper plots, solid red lines represent the associations derived from the distributed lag non-linear models, expressed as beta coefficients of the crown-rump length with their respective 95% confidence intervals in grey. Coefficients are estimated as the change in crown-rump length in millimeters at each temperature respective to the reference temperature (black dotted lines, 22.4 ºC for **A**, and 9.0 ºC for **B**). Blue and red shaded areas (exposure to colder or warmer temperatures respective to the reference temperature) indicate associations surviving correction for multiple testing (*P* < 0.025). The lower plots represent the distribution of temperatures, with the blue, green, and red dotted lines indicating the 5^th^ percentile, mean, and 95^th^ percentile of temperature distribution, respectively. Distributed lag non-linear models were adjusted for fetal biological sex; parental age at recruitment, national origin, educational level, and body mass index; parents partnered or co-habiting, monthly net household income, maternal parity, alcohol consumption, smoking habit, folic acid use, and month of last menstrual period; residential surrounding greenness, neighborhood socioeconomic status, and gestational age at the ultrasound. Abbreviations: CI, confidence interval. |

| **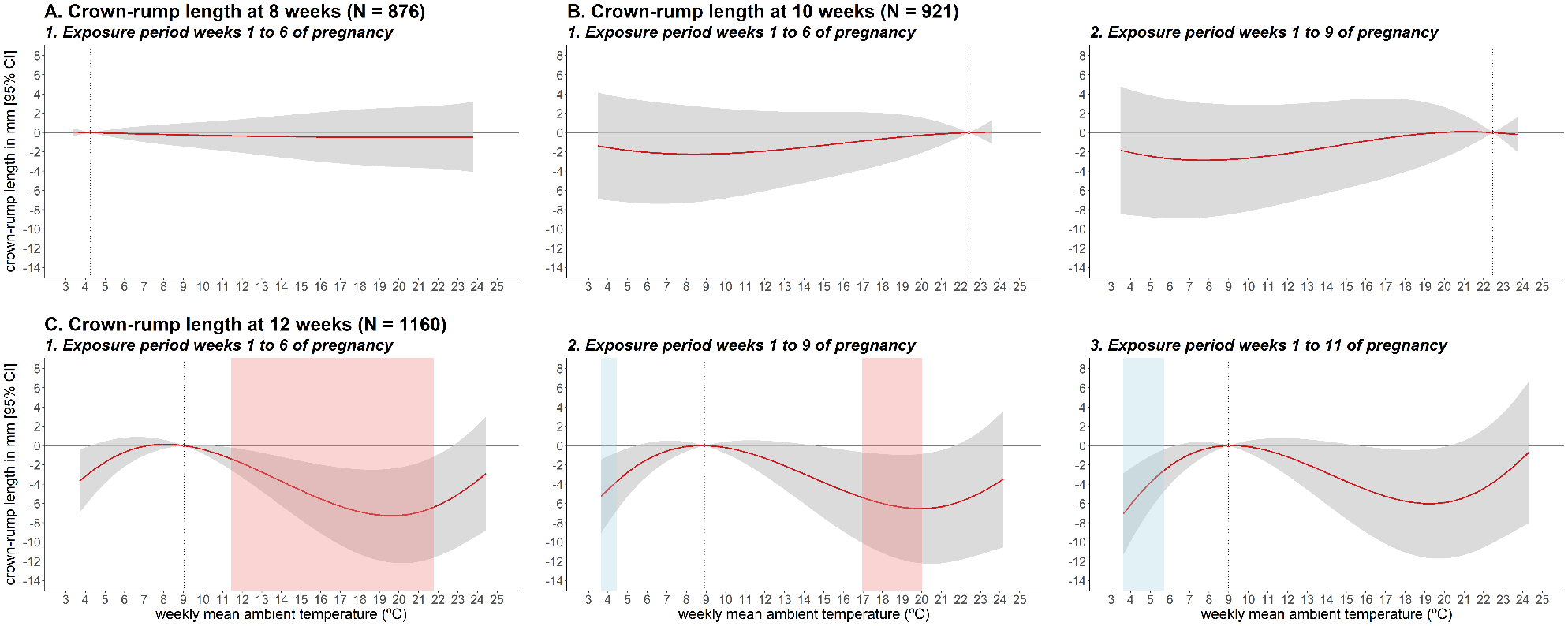** |
| --- |
| **Figure S11. Cumulative associations between weekly ambient temperature exposure at different lag periods and crown-rump length at 8, 10, and 12 weeks of pregnancy, with the DLNM exposure-response relationship modelled with knots at the 10^th^ and 90^th^ percentile of temperature distribution.**  The x-axes depict the 3^rd^ to 97^th^ percentiles of the temperature distribution. In the upper plots, solid red lines represent the associations derived from the distributed lag non-linear models, expressed as beta coefficients of the crown-rump length with their respective 95% confidence intervals in grey. Coefficients are estimated as the change in crown-rump length in millimetres at each temperature respective to the reference temperature (black dotted lines). Blue and red shaded areas (exposure to colder or warmer temperatures respective to 8.9 ºC) indicate statistically significant associations after correction for multiple testing (*P* < 0.025). Distributed lag non-linear models were adjusted for child biological sex; parental age at recruitment, national origin, educational level, and body mass index; parents partnered or co-habiting, monthly net household income, maternal parity, alcohol consumption, smoking habit, folic acid use, and month of last menstrual period; residential surrounding greenness, neighbourhood socioeconomic status, and gestational age at the ultrasound. Abbreviations: CI, confidence interval. |
| **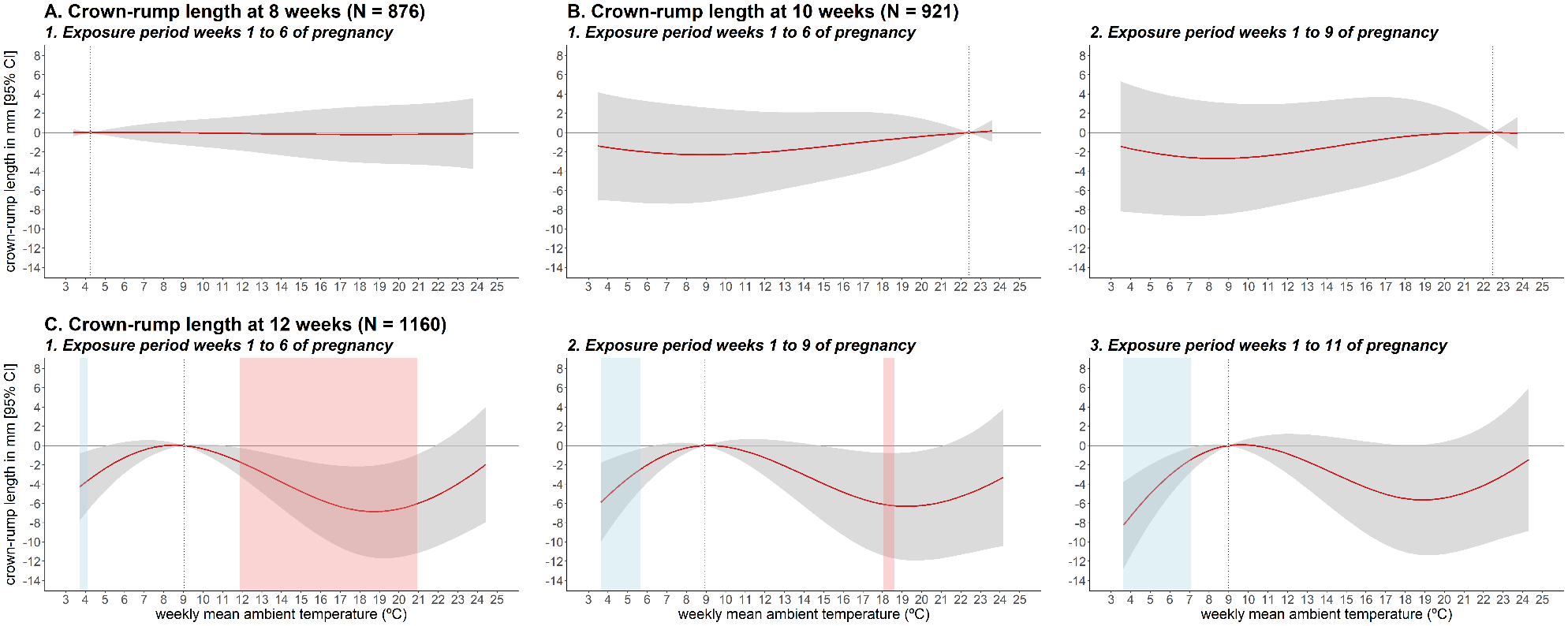** |
| **Figure S12. Cumulative associations between weekly ambient temperature exposure at different lag periods and crown-rump length at 8, 10, and 12 weeks of pregnancy, with the DLNM lag-response relationship modelled with one knot at the median lag on the log-scale.**  The x-axes depict the 3^rd^ to 97^th^ percentiles of the temperature distribution. In the upper plots, solid red lines represent the associations derived from the distributed lag non-linear models, expressed as beta coefficients of the crown-rump length with their respective 95% confidence intervals in grey. Coefficients are estimated as the change in crown-rump length in millimetres at each temperature respective to the reference temperature (black dotted lines). Blue and red shaded areas (exposure to colder or warmer temperatures respective to 8.9 ºC) indicate statistically significant associations after correction for multiple testing (*P* < 0.025). Distributed lag non-linear models were adjusted for child biological sex; parental age at recruitment, national origin, educational level, and body mass index; parents partnered or co-habiting, monthly net household income, maternal parity, alcohol consumption, smoking habit, folic acid use, and month of last menstrual period; residential surrounding greenness, neighbourhood socioeconomic status, and gestational age at the ultrasound. Abbreviations: CI, confidence interval. |
| **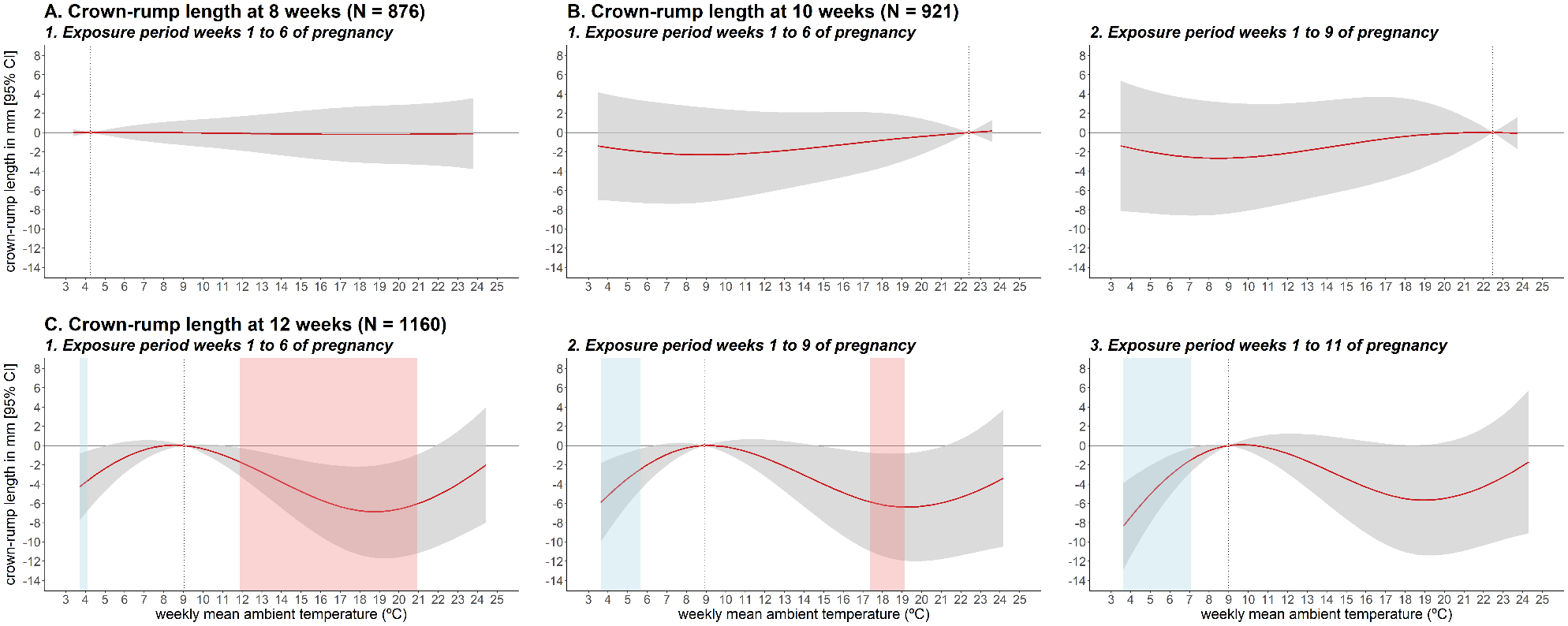** |
| **Figure S13. Cumulative associations between weekly ambient temperature exposure at different lag periods and crown-rump length at 8, 10, and 12 weeks of pregnancy, with the DLNM lag-response relationship modelled with one knot at the median lag equally spaced.**  The x-axes depict the 3^rd^ to 97^th^ percentiles of the temperature distribution. In the upper plots, solid red lines represent the associations derived from the distributed lag non-linear models, expressed as beta coefficients of the crown-rump length with their respective 95% confidence intervals in grey. Coefficients are estimated as the change in crown-rump length in millimetres at each temperature respective to the reference temperature (black dotted lines). Blue and red shaded areas (exposure to colder or warmer temperatures respective to 8.9 ºC) indicate statistically significant associations after correction for multiple testing (*P* < 0.025). Distributed lag non-linear models were adjusted for child biological sex; parental age at recruitment, national origin, educational level, and body mass index; parents partnered or co-habiting, monthly net household income, maternal parity, alcohol consumption, smoking habit, folic acid use, and month of last menstrual period; residential surrounding greenness, neighbourhood socioeconomic status, and gestational age at the ultrasound. Abbreviations: CI, confidence interval. |
| **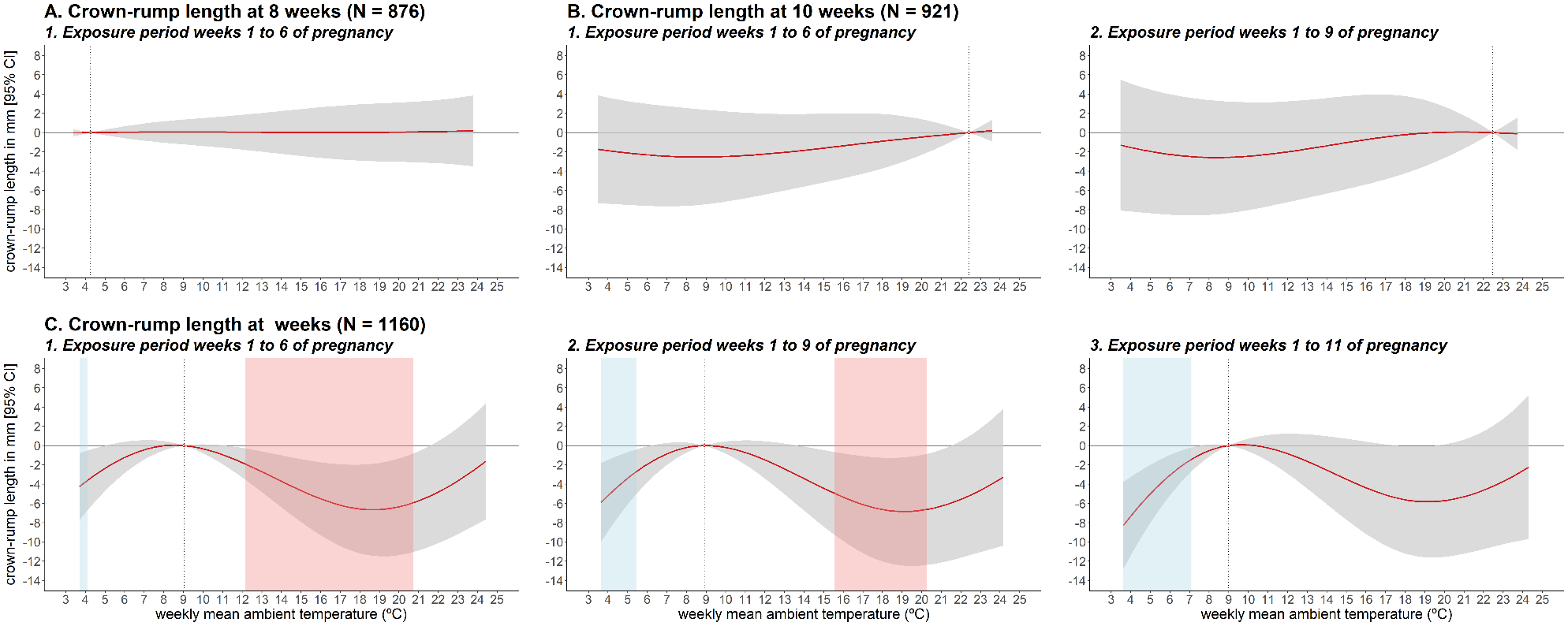** |
| **Figure S14. Cumulative associations between weekly ambient temperature exposure at different lag periods and crown-rump length at 8, 10, and 12 weeks of pregnancy, with the DLNM lag-response relationship modelled with two equally spaced knots.**  The x-axes depict the 3^rd^ to 97^th^ percentiles of the temperature distribution. In the upper plots, solid red lines represent the associations derived from the distributed lag non-linear models, expressed as beta coefficients of the crown-rump length with their respective 95% confidence intervals in grey. Coefficients are estimated as the change in crown-rump length in millimetres at each temperature respective to the reference temperature (black dotted lines). Blue and red shaded areas (exposure to colder or warmer temperatures respective to 8.9 ºC) indicate statistically significant associations after correction for multiple testing (*P* < 0.025). Distributed lag non-linear models were adjusted for child biological sex; parental age at recruitment, national origin, educational level, and body mass index; parents partnered or co-habiting, monthly net household income, maternal parity, alcohol consumption, smoking habit, folic acid use, and month of last menstrual period; residential surrounding greenness, neighbourhood socioeconomic status, and gestational age at the ultrasound. Abbreviations: CI, confidence interval. |

| **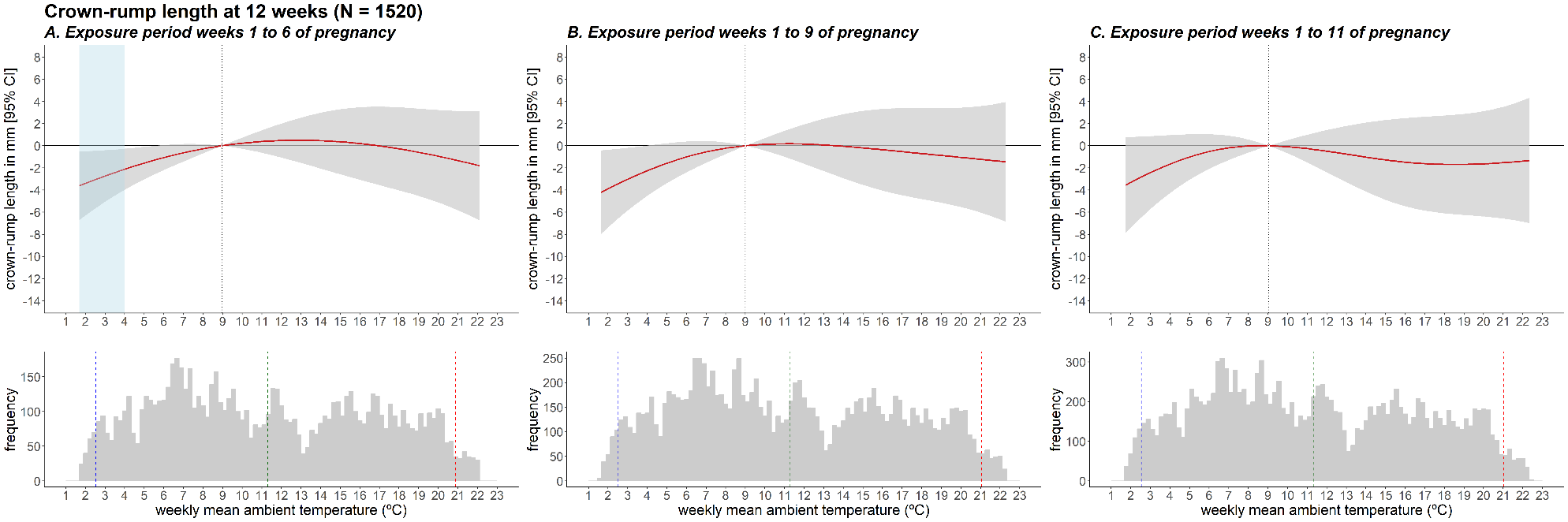** |
| --- |
| **Figure S15. Cumulative associations between weekly ambient temperature exposure for different exposure periods and crown-rump length at 12 weeks of pregnancy in the replication cohort Generation R.**  The x-axes depict the 3^rd^ to 97^th^ percentiles of the temperature distribution. Solid red lines represent the associations derived from the distributed lag non-linear models, expressed as beta coefficients of the crown-rump length with their respective 95% confidence intervals in grey. Coefficients are estimated as the change in crown-rump length in millimetres at each temperature respective to the reference temperature (black dotted lines, 9.0 ºC). Blue shaded areas (exposure to colder temperatures respective to 8.9 ºC) indicate statistically significant associations after correction for multiple testing (*P* < 0.025). The lower plots represent the distribution of temperatures, with the blue, green, and red dotted lines indicating the 5^th^ percentile, mean, and 95^th^ percentile of temperature distribution, respectively. Distributed lag non-linear models were adjusted for child biological sex; parental age at recruitment, national origin, and body mass index; marital status of parents, monthly net household income, maternal education level, parity, alcohol consumption, smoking habit, folic acid use, and month of last menstrual period; residential surrounding greenness, neighbourhood socioeconomic status, and gestational age at the ultrasound. Abbreviations: CI, confidence interval. |

| **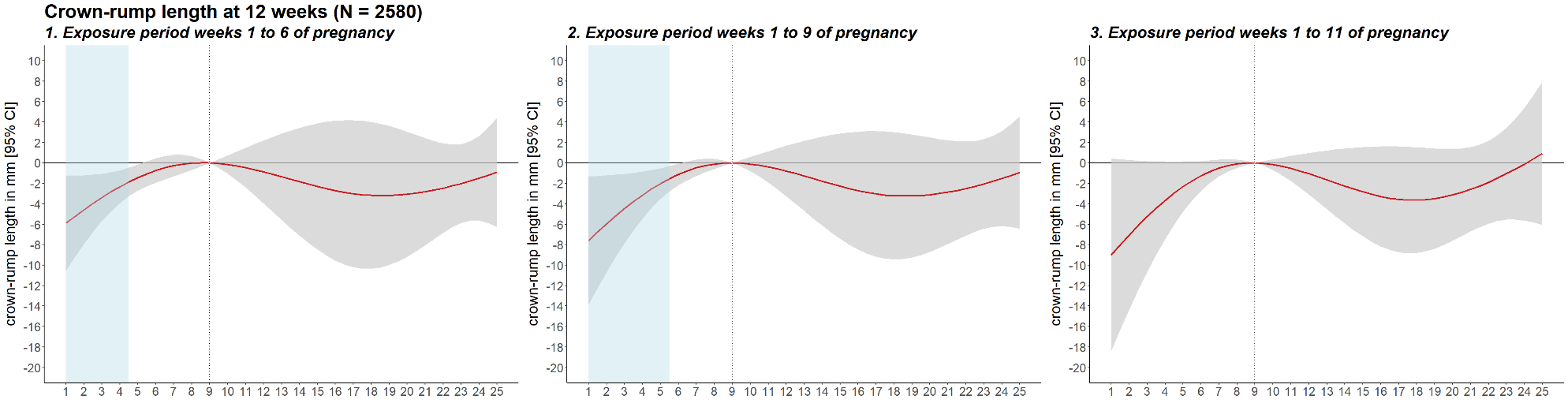** |
| --- |
| **Figure S16. Cumulative associations between weekly ambient temperature exposure for different exposure periods and crown-rump length at 12 weeks of pregnancy in the primary cohort Generation R Next and the replication cohort Generation R.**  The x-axes depict the 3^rd^ to 97^th^ percentiles of the temperature distribution. Solid red lines represent the associations derived from the random-effects meta-analysis, expressed as beta coefficients of the crown-rump length with their respective 95% confidence intervals in grey. Coefficients are estimated as the change in crown-rump length in millimetres at each temperature respective to the reference temperature (black dotted lines, 9.0 ºC). Blue shaded areas (exposure to colder temperatures respective to 9.0 ºC) indicate statistically significant associations after correction for multiple testing (*P* < 0.025). Within each cohort, distributed lag non-linear models were adjusted for child biological sex; parental age at recruitment, national origin, and body mass index; marital status of parents, monthly net household income, maternal education level, parity, alcohol consumption, smoking habit, folic acid use, and month of last menstrual period; residential surrounding greenness, neighbourhood socioeconomic status, and gestational age at the ultrasound. Abbreviations: CI, confidence interval. |

**References**

Annoni, A., Luzet, C., Gubler, E., & Ihde, J. (2001). *Map Projections for Europe*.

Corpernicus. (n.d.). *CORINE Land Cover*. Retrieved November 29, 2022, from https://land.copernicus.eu/pan-european/corine-land-cover

De Ridder, K., Lauwaet, D., & Maiheu, B. (2015). UrbClim - A fast urban boundary layer climate model. *Urban Climate*, *12*. https://doi.org/10.1016/j.uclim.2015.01.001

ECMWF. (n.d.). *ERA5*. Retrieved November 29, 2022, from https://www.ecmwf.int/en/forecasts/datasets/reanalysis-datasets/era5

Essers, E., Granés, L., Delaney, S., Ballester, J., Santos, S., Petricola, S., Yang, T. C., Fernández-Somoano, A., Bereziartua, A., Ballester, F., Tardón, A., Vrijheid, M., Lertxundi, A., McEachan, R. R. C., El Marroun, H., Tiemeier, H., Iñiguez, C., & Guxens, M. (2024). Ambient air temperature exposure and foetal size and growth in three European birth cohorts. *Environment International*, *186*, 108619. https://doi.org/10.1016/j.envint.2024.108619

Essers, E., Kusters, M., Granés, L., Ballester, J., Petricola, S., Lertxundi, N., Arregi, A., Ballester, F., Vrijheid, M., El Marroun, H., Iñiguez, C., Tiemeier, H., & Guxens, M. (2025). Temperature Exposure and Psychiatric Symptoms in Adolescents From 2 European Birth Cohorts. *JAMA Network Open*, *8*(1), e2456898. https://doi.org/10.1001/jamanetworkopen.2024.56898

García-Díez, M., Lauwaet, D., Hooyberghs, H., Ballester, J., De Ridder, K., & Rodó, X. (2016). Advantages of using a fast urban boundary layer model as compared to a full mesoscale model to simulate the urban heat island of Barcelona. *Geoscientific Model Development*, *9*(12). https://doi.org/10.5194/gmd-9-4439-2016

Golan, R., Kloog, I., Almog, R., Gesser-Edelsburg, A., Negev, M., Jolles, M., Shalev, V., Eisenberg, V. H., Koren, G., Abu Ahmad, W., & Levine, H. (2018). Environmental exposures and fetal growth: the Haifa pregnancy cohort study. *BMC Public Health*, *18*(1). https://doi.org/10.1186/s12889-018-5030-8

Granés, L., Essers, E., Ballester, J., Petricola, S., Tiemeier, H., Iñiguez, C., Soriano-Mas, C., & Guxens, M. (2024). Early life cold and heat exposure impacts white matter development in children. *Nature Climate Change*, *14*(7), 760–766. https://doi.org/10.1038/s41558-024-02027-w

Granés, L., Kusters, M. S. W., Ballester, J., Essers, E., Petricola, S., López-Vicente, M., Iñiguez, C., Tiemeier, H., Muetzel, R. L., Soriano-Mas, C., & Guxens, M. (2025). Exposure to Ambient Temperature and Functional Connectivity of Brain Resting-State Networks in Preadolescents. *Journal of the American Academy of Child & Adolescent Psychiatry*. https://doi.org/10.1016/j.jaac.2024.11.023

Honaker, J., King, G., & Blackwell, M. (2012). *AMELIA II: A Program for Missing Data, Version 1.6.2*.

Lauwaet, D., De Ridder, K., Saeed, S., Brisson, E., Chatterjee, F., van Lipzig, N. P. M., Maiheu, B., & Hooyberghs, H. (2016). Assessing the current and future urban heat island of Brussels. *Urban Climate*, *15*. https://doi.org/10.1016/j.uclim.2015.11.008

Lauwaet, D., Hooyberghs, H., Maiheu, B., Lefebvre, W., Driesen, G., Van Looy, S., & De Ridder, K. (2015). Detailed urban heat island projections for cities worldwide: Dynamical downscaling CMIP5 global climate models. *Climate*, *3*(2). https://doi.org/10.3390/cli3020391

NASA. (n.d.). *MODIS Vegetation Index Products (NDVI and EVI)*. Retrieved November 29, 2022, from https://modis.gsfc.nasa.gov/data/dataprod/mod13.php
